# Spatial profiling of HPV-stratified head and neck squamous cell carcinoma reveals distinct immune niches and microenvironmental architectures

**DOI:** 10.1186/s12967-025-07280-x

**Published:** 2025-11-18

**Authors:** Ettai Markovits, Dmytro Klymyshyn, Roni Froumine, Hailing Zong, Michael Mints, Sangeetha Mahadevan, Kenneth Bloom, Jamie Bates, Gareth J. Thomas, Lauri Diehl, Oscar Puig, Abhishek Aggarwal

**Affiliations:** 1 Nucleai, Tel Aviv, Israel; 2 Gilead Sciences Inc., Foster City, CA USA; 3 University of Southampton, Southampton, UK

## Abstract

**Background:**

HPV status is a key determinant of prognosis and treatment response in head and neck squamous cell carcinoma (HNSCC). To investigate how HPV influences the tumor-immune-stromal landscape, we performed high-dimensional spatial profiling, including its impact on spatial organization, tertiary lymphoid structures (TLSs), and spatially organized cellular neighborhoods.

**Methods:**

Tumor biopsies from HNSCC patients (*n* = 16; 7 HPV-positive, 9 HPV-negative) were stained with a multiplex immunofluorescence (mIF) panel focused on immune profiling. A deep learning-based analysis pipeline enabled the identification and phenotypic state profiling of 14 cell types. Tissues were segmented into four distinct tumor regions, and spatial neighborhoods and TLSs were identified and analyzed for differential cellular composition, activation states, and spatial interactions between HPV-positive and HPV-negative tumors.

**Results:**

HPV-positive and HPV-negative tumors differ in their tumor microenvironment (TME) composition, tumor cell state and spatial organization. The TME of HPV-positive tumors exhibited a greater abundance of activated lymphocytes, B- and T-cell-enriched spatial neighborhoods, and PD-1–PD-L1 interactions within the tumor area, whereas HPV-negative tumors were dominated by fibroblast- and macrophage-rich niches. *T*- cells in HPV-positive tumors showed greater activation across neighborhoods and areas, while in HPV-negative tumors *T*- cells demonstrated enrichment of exhaustion and terminal differentiation markers such as PD-1 and CD57. HPV-positive tumor cells had increased IDO1, HLA-DR, and Ki67 positivity, whereas HPV-negative tumor cells were more frequently CD44 positive, reflecting a more stem-like phenotype. Importantly, TLSs in HPV-positive tumors were located closer to the tumor area and enriched in activated immune cells, including ICOS^+^ CD4 *T*- cells, memory *T*- cells, and CD21^+^ B- cells. In contrast, TLSs in HPV-negative tumors were more distant and enriched for immunosuppressive populations such as PD-1^+^/PD-L1^+^ Tregs and macrophages.

**Conclusions:**

HPV status defines distinct spatial immune architectures in HNSCC. HPV-positive tumors harbor immune-activating TLSs and cellular neighborhoods that support antitumor immunity, whereas HPV-negative tumors exhibit suppressive niches and stromal dominance. These findings highlight TLSs, particularly their proximity and composition, as key features of the HPV-stratified TME and potential biomarkers for immunotherapy response.

**Graphical abstract:**

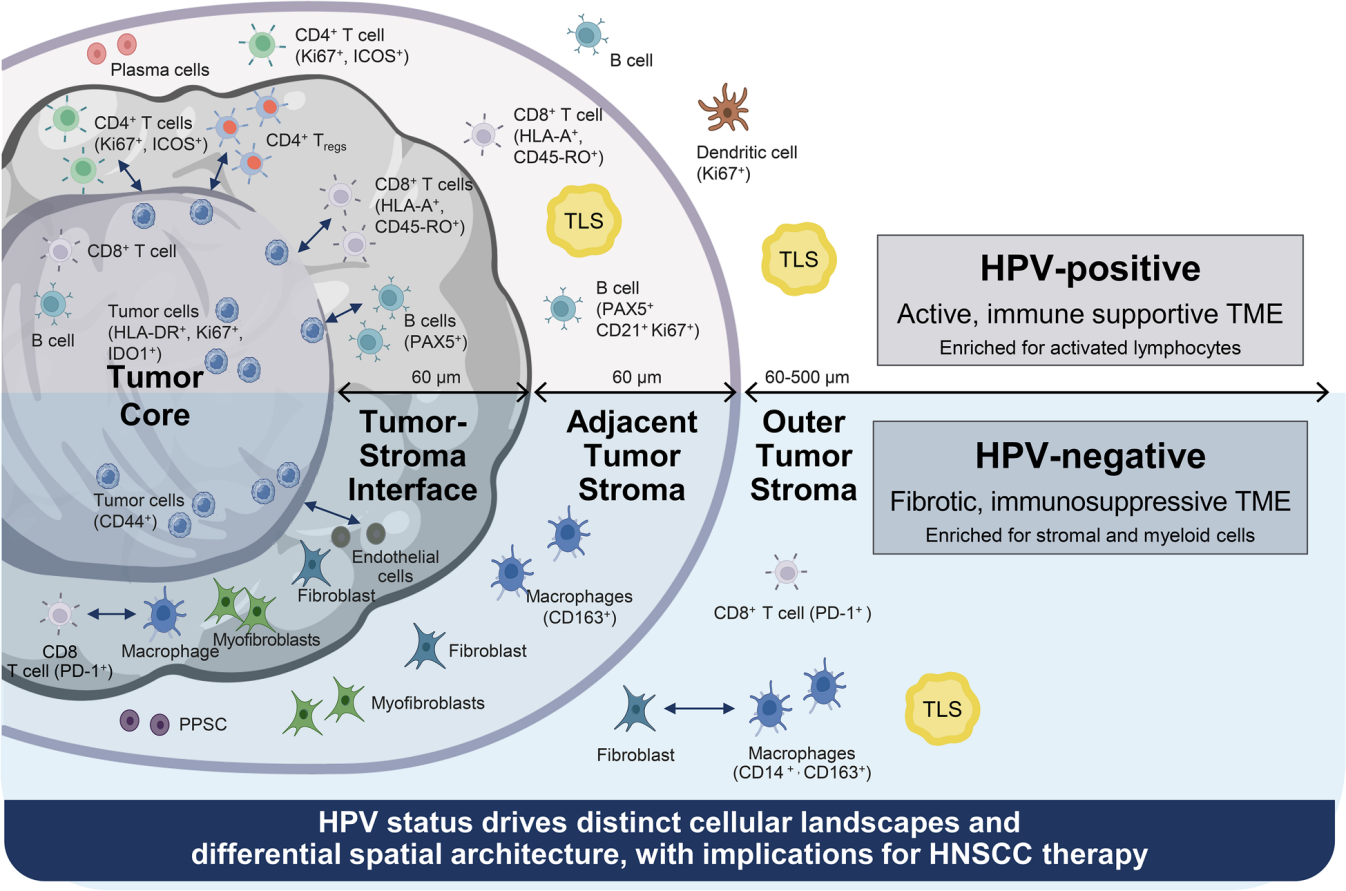

**Supplementary information:**

The online version contains supplementary material available at 10.1186/s12967-025-07280-x.

## Background

Head and neck squamous cell carcinoma (HNSCC) is the seventh leading form of cancer worldwide, accounting for 450,000 cancer deaths worldwide [1]. HNSCC is more common in men and arises from two distinct etiological pathways: one driven by human papillomavirus (HPV) infection and the other by exposure to carcinogens such as tobacco and alcohol [2]. These two etiologies give rise to histologically and clinically distinct disease subtypes. Compared with HPV-negative tumors, HPV-positive tumors are associated with a better response to therapy and significantly improved survival outcomes [3, 4].

The tumor microenvironment (TME) encompasses the nonneoplastic cellular components present within and surrounding the tumor, including immune cells, fibroblasts, endothelial cells, and other cell types, all of which are embedded in the tumor stroma [5]. The composition and abundance of these cell types define the TME and play critical roles in modulating tumor behavior and the response to therapy. Recent studies have shown that, in addition to the mere presence and density of cell types, the spatial organization of the TME serves as a critical prognostic factor influencing survival and therapeutic response in multiple cancer types [6–8]. Over the past decade, advanced spatial profiling techniques have been developed to characterize the spatial architecture of tumor-TME interactions in detail. Among these methods, multiplex immunofluorescence (mIF) has emerged as a pivotal tool, offering insights into patient response and resistance [9].

Research on the spatial architecture of the TME in HNSCC has demonstrated that in HPV-negative HNSCC, patients whose primary tumors exhibit greater compartmentalization between neoplastic tumor cells and immune cell populations experience longer progression-free survival (PFS) [10]. This effect is attributed to higher densities of immune cells in compartmentalized tumor regions and, conversely, greater densities of neoplastic tumor cells in mixed tumor regions. Furthermore, CD8 and CD4 *T*- cells in mixed tumor regions exhibit exhausted phenotypes characterized by elevated PD-1 expression. These studies provided insights into spatial contexture differences between HPV-positive and HPV-negative patients: HPV-positive tumors have greater infiltration of immune cells, including higher densities of CD8^+^ and CD4^+^ T lymphocytes, and of CD68^+^ and CD68^+^ CD163^+^ macrophages [11–13]. These tumors also show closer spatial proximity to CD8^+^ T lymphocytes, with a greater percentage of tumor cells within a 20 µm radius of a CD8^+^ T-cell and an increased density of PD-L1^+^ tumor cells, and all these features are correlated with disease-free survival [13]. Muijlwijk and colleagues recently provided spatial characterization of the TME in treatment-naive HNSCC tumors using mIF and secretome profiling. They identified distinct immunotypes based on CD8+ T cell infiltration and revealed site-specific differences in immune cell composition, localization, and interactions, particularly in HPV stratified oropharyngeal squamous cell carcinoma (OPSCC) highlighting immune cell differences HPV-positive OPSCC showing higher B and T lymphocyte infiltration compared to HPV-negative cases [12]. Spatial transcriptomics corroborated these findings, indicating that HPV-positive HNSCC tumors are infiltrated by lymphoid cells, whereas HPV-negative tumors exhibit increased macrophage densities, with a central role for the ephrin-A (EPHA2) pathway [14]. In HPV-negative HNSCC, interactions between cancer cells and the surrounding TME, specifically, fibroblast-enriched stroma or immune cell infiltrates at the tumor margin, are associated with poor and favorable prognoses, respectively [12, 15]. Recent advances have enabled accurate survival prediction in HPV-negative HNSCC patients by analyzing the identified local spatial contexts [12, 16].

While the TME is a key regulator of tumor progression, immune escape, and therapeutic resistance, tertiary lymphoid structures (TLSs) have gained recognition as crucial mediators of antitumor immunity across various malignancies, including HNSCC [17–20]. TLSs contribute to immune activation by supporting dendritic cell-driven antigen presentation, fostering B and *T*- cell activation, and maintaining *T*- cell functionality in settings of persistent antigen exposure [21]. The presence of TLSs in the TME has been associated with enhanced responses to immunotherapy and improved patient survival [18], highlighting their potential as predictive biomarkers of treatment efficacy. Accumulating evidence indicates that the presence of TLSs alone does not fully explain clinical outcomes. Factors such as TLS architecture and cellular constituents appear to significantly impact immunological function, particularly in facilitating antigen presentation and immune cell infiltration [21]. A recent study by Sadeghirad and colleagues emphasized the relevance of TLS spatial distribution, revealing that proximity to tumor cells offers critical insights into their role in modulating antitumor responses and influencing therapeutic success [20]. These findings underscore the importance of further elucidating the spatial and functional properties of TLSs to better leverage them as biomarkers and targets in cancer immunotherapy.

Understanding the structure and spatial organization of cells in the HNSCC TME and how HPV infection alters this structure is crucial for identifying predictive biomarkers of response to therapy and, in the context of HPV-positive disease, for stratifying patients who may benefit from treatment de-escalation strategies. To this end, we utilized mIF to analyze tumor samples from HPV-positive and HPV-negative HNSCC patients and identified structural features in the tumor and TME that distinguish both of these classes of tumors. Our findings provide valuable insights into HNSCC tumor structure and confirm that HPV status is a significant factor impacting tumor architecture.

## Methods

### Human biopsies

This study included formalin-fixed, paraffin-embedded (FFPE) tumor tissues from two cohorts of patients. The first cohort comprised 10 patients who underwent surgical tumor resection at Poole Hospital (Poole, Dorset, UK), with ethical approval from the UK National Research Ethics Service and informed consent obtained from all participants [22]. HPV status was confirmed using p16 immunohistochemistry as part of the standard of care in a clinical NHS histopathology lab at Poole Hospital NHS Foundation Trust. This was combined with assessing HPV-encoded gene expression using scRNASeq data from each case by aligning and mapping reads with a human-HPV hybrid reference genome as previously described [22]. 7 patients out of 10 were confirmed HPV-positive from this cohort. The second cohort of 6 HNSCC FFPE blocks was obtained from commercial vendors (Cureline, BioIVT) and collected under appropriate ethical approval and patient consent for research use. The HPV status of these samples was confirmed by PCR-based detection of HPV L1 DNA using primer pair: MY09 (Forward Primer): CGT CCM ARR GGA WAC TGA TC; MY11 (Reverse Primer): GCM CAG GGW CAT AAY AAT GG. Detailed sample information is provided in Suppl. Table S1.

### Multiplex immunofluorescence (mIF) using PhenoCycler-fusion

Staining and whole-slide imaging were performed using the PhenoCycler-Fusion (Akoya Biosciences, USA). The reader is referred to Supplementary Methods for additional information.

### Cell typing and marker quantification

Whole-slide images (WSIs) were analyzed using NucleAI’s mIF analysis pipeline [23]. The reader is referred to Supplementary Methods for additional information.

### H&E-based tissue segmentation

A convolutional neural network (CNN) was trained on 190 expert-annotated regions of interest (ROIs) to segment tissue into four categories: tumor, tumor-stroma, necrosis, and other tissue types. The reader is referred to Supplementary Methods for additional information.

### Cellular neighborhood identification

Cellular neighborhoods were identified as previously described [7]. The reader is referred to Supplementary Methods for additional information.

### Tertiary lymphoid structures (TLSs)

TLSs were identified through a multistep analysis. Germinal centers (GCs) were first defined as regions ≥500 µm^2^ in area with a high density of CD21^+^ B- cells and dendritic cells. Lymphoid aggregates were then identified as regions enriched with B- cells, CD4 *T*- cells, dendritic cells, and plasma cells. To define TLSs, GCs were expanded by a 100 µm radius to include surrounding stromal components and intersected with lymphoid aggregates. Regions resulting from this intersection that exceeded 20,000 µm^2^ were classified as TLSs. To exclude mature lymphoid structures unrelated to the tumor, TLSs located more than 1,000 µm from the nearest tumor region were excluded from downstream analyses. TLSs were defined as tumor-adjacent (ta-TLS) if the mean distance between the cells within the TLS and the nearest tumor area was less than 200 µm and as distant (d-TLS) if the mean distance was greater than 200 µm.

### Spatial feature calculation

Spatial features were calculated to capture cell distributions, cellular states (cell type combined with marker positivity), and cell‒cell interactions. These features included cell type and cell state fractions from all cells within a compartment (area, neighborhood or a TLS) and cell state enrichment within cells in a compartment. Cell-cell interactions and receptor-ligand interactions, such as PD-1/PD-L1, were quantified by calculating the fraction of cells with at least one neighboring cell within 25 µm, based on distances measured between cell centroids measured within a given area. The reader is referred to Supplementary Methods for additional information.

### Statistical analysis

The Mann‒Whitney U test was used to identify the differential distributions of spatial features and primary components. Fisher’s exact test was used to assess the differential distribution of categorical variables. Where specified, multiple testing correction was applied using the Benjamini–Hochberg procedure. All adjusted p-values are reported in the relevant supplementary tables. PCA was conducted on the 500 most variable normalized spatial features, following the filtration of highly correlated features. To assess the biological representation of each primary component, the Pearson correlation coefficient was calculated between the top 10 primary components and the spatial features.

## Results

### Spatial profiling of HPV-positive and HPV-negative HNSCC by multiplex immunofluorescence

**Fig. 1:**
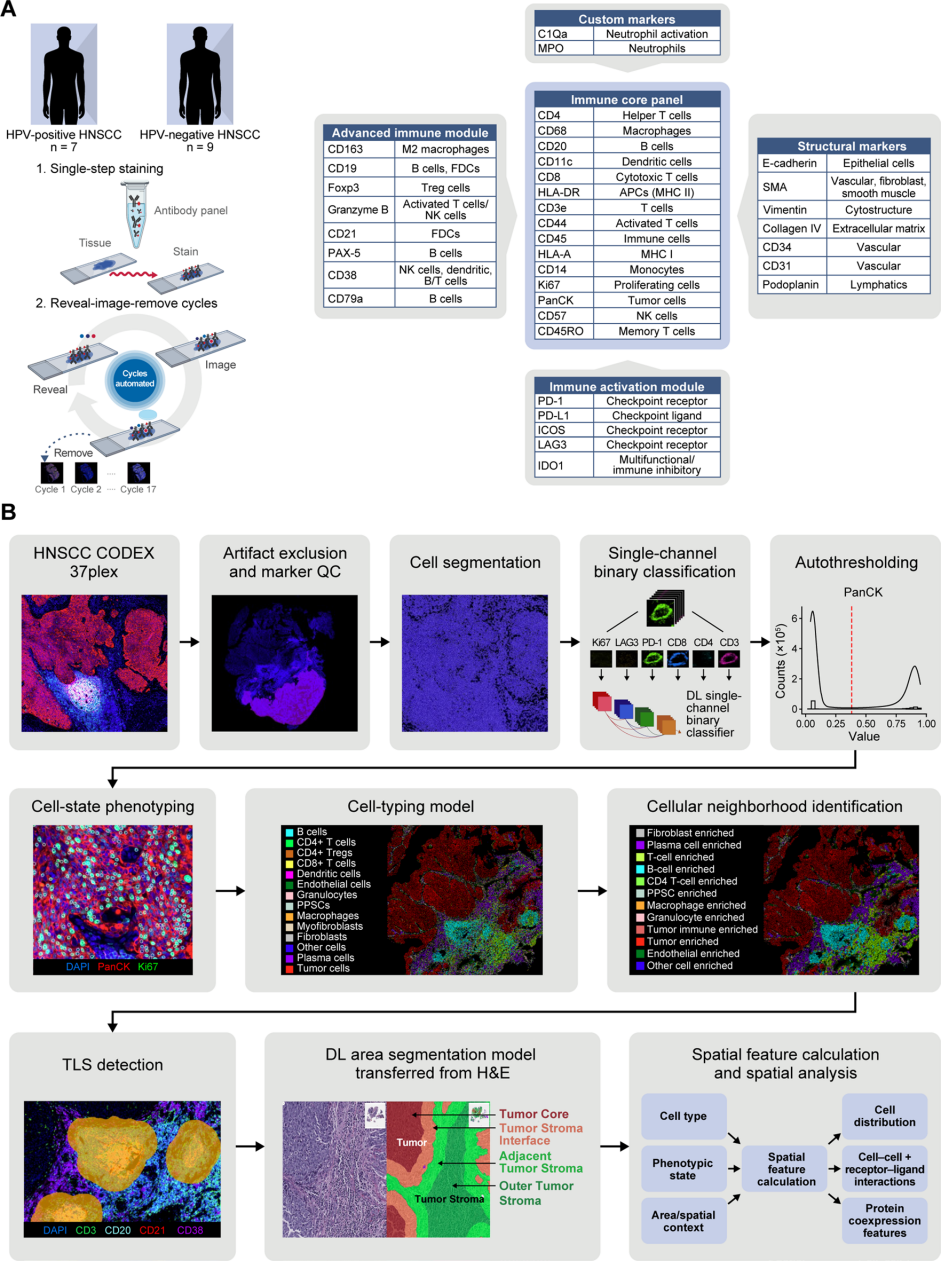
Overview of multiplex immunofluorescence (mIF) image generation and the analytical pipeline. (**A**) Schematic representation of the mIF staining and imaging process (left) and the 37-antibody panel designed to target tumor, immune, and stromal cell markers, along with immune phenotypic state markers (right). (**B**) Analysis pipeline - following channel and image QC, including large-staining defect removal, we performed cell segmentation followed by DL-based protein positivity classification for each marker within each cell. These outputs were used to determine the cell type and cell state of each cell, followed by spatial neighborhood and area assignment, including tertiary lymphoid structures, to contextualize each cell within its spatial environment. Finally, we combined the cell type, phenotypic state, area and neighborhood assignments to calculate human-interpretable spatial features, which can be used to derive actionable spatial insights

To investigate the different spatial compositions of HPV-positive and HPV-negative HNSCC tumors, formalin-fixed paraffin-embedded (FFPE) sections of 16 tumors from 7 HPV-positive and 9 HPV-negative HNSCC patients (Suppl. Table S1) were stained with an mIF panel containing 37 antibodies, which included tumor, immune and stromal cell markers, as well as immune phenotypic state markers (Fig. 1A). Whole-slide images (WSIs) were generated after mIF staining, and quality control (QC) was performed to remove out of focus areas, staining artifacts and tissue folds. WSI areas that passed the QC were analyzed downstream (Fig. 1B).

Using a deep learning-based mIF analytical pipeline, > 2.5 million cells were segmented, and marker positivity was established for each detected cell (Fig. 1B). On the basis of known marker expression profiles, 14 distinct cell types were identified, including B- cells, CD4^+^ *T*- cells, CD4^+^ T_regs_, CD8^+^ *T*- cells, dendritic cells, endothelial cells, fibroblasts, granulocytes, podoplanin-positive stromal cells (PPSCs), macrophages, myofibroblasts, plasma cells, tumor cells and marker-negative (“other”) cells (Fig. 1Band Suppl. Figure S1A, Suppl. Table S2A). The supervised cell typing approach demonstrated high accuracy in comparison with expert annotations (balanced accuracy > 80% in all cell types and F1 scores over 75% in > 85% of the cell types, Suppl. Figure S1B) and high concordance with unsupervised clustering of positive prediction probabilities (Suppl. Figure S1C).

To delineate distinct tissue compartments within the tumor biopsy, same-slide hematoxylin and eosin (H&E) stained images were analyzed using a deep learning-based area segmentation model (Fig. 1B, Suppl. Figure S1D). The tumor and tumor-stroma compartments were further subdivided into four spatially defined regions (Fig. 1B): 1) the tumor core (TC), which is the tumor parenchyma with tumor nests, excluding the tumor region 60 µm closest to the tumor/stroma interface; 2) the tumor-stroma interface (TSI), which is the 60 µm tumor area adjacent to the tumor stroma; 3) the adjacent tumor stroma (aTS), which is the region in the TME 60 µm from the tumor stroma; and 4) the outer tumor stroma (oTS), which is the region in the stroma within 500 µm from the tumor stromal interface, excluding the aTS.

### Differential cell composition and activation states in the tumor stroma of HPV-positive and HPV-negative tumors

**Fig. 2:**
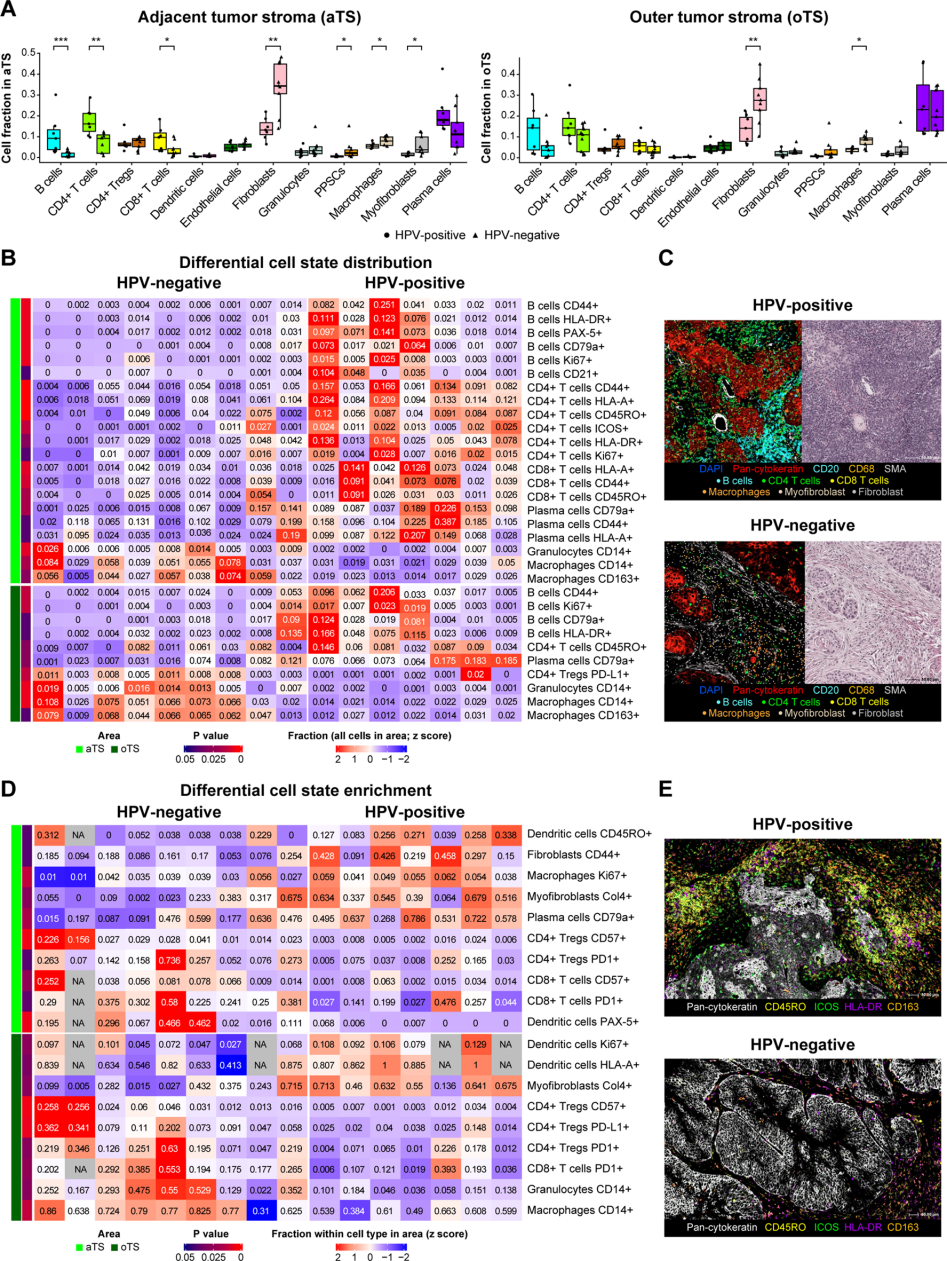
Differential cell composition and activation states in the tumor stroma of HPV-positive and HPV-negative HNSCC tumors. (**A**) Box plots demonstrating differential cell type fraction distributions in adjacent tumor-stroma (aTS, left) and outer tumor-stroma (oTS, right) regions of HPV-positive (*n* = 7) and HPV-negative (*n* = 9) tumors. (**B**) Heatmap illustrating differentially distributed cell state fractions between HPV-positive (*n* = 7) and HPV-negative (*n* = 9) tumors in the aTS and oTS. (**C**) Representative mIF (left) and H&E (right) images of HPV-positive and HPV-negative tumors, illustrating lymphocytes, stroma and macrophages. (**D**) Heatmap illustrating differential cell state enrichment between HPV-positive (*n* = 7) and HPV-negative (*n* = 9) tumors in the aTS and oTS. (**E**) Representative mIF images of HPV-positive and HPV-negative tumors, illustrating the differential distribution of cell states within lymphocytes and macrophages in the tumor stroma. *p* values were calculated using the Mann‒Whitney U test. * = *p* < 0.05, ** = *p* < 0.01, *** = *p* < 0.001. PPSC; podoplanin-positive stromal cells

To characterize the spatial organization and activation states of immune cells further, we profiled the adjacent tumor stroma (aTS) and outer tumor stroma (oTS, Fig. 2A).

In the aTS, HPV-positive tumors presented a significantly greater abundance of lymphocytes, including B- cells (mean cell fraction: 10.9% in the HPV-positive vs. 1.3% in HPV-negative, **p = 0.0007**), CD4^+^ *T*- cells (17.5% vs. 7.2%; **p = 0.007**), and CD8^+^ *T*- cells (8.9% vs. 3.7%; **p = 0.03**). In contrast, HPV-negative tumors were enriched with macrophages (5.6% vs. 8.0%; **p = 0.02**) and stromal cell types, including fibroblasts (13.8% vs. 33.9%; **p = 0.003**), myofibroblasts (1.5% vs. 5.6%; **p = 0.02**), and PPSCs (0.6% vs. 3.5%; **p = 0.04**) (Fig. 2A and Suppl. Table S3A). Further analysis of the cell state distribution by studying co-expressing activation and functional markers within the aTS revealed that HPV-positive tumors harbored a greater fraction of activated lymphocytes, characterized by CD44^+^ and HLA-A^+^ expression across B- cells, CD4^+^ *T*- cells, CD8^+^ *T*- cells, and plasma cells (Fig. 2B–C). Additionally, memory phenotypes (CD45RO^+^) of CD4^+^ and CD8^+^ *T*- cells, as well as ICOS^+^ CD4^+^ *T*- cells, were more prevalent in HPV-positive tumors. Conversely, HPV-negative tumors presented a greater abundance of CD14^+^ macrophages and granulocytes, along with M2-like macrophages expressing CD163 (Fig. 2B–C, Suppl. Table S4B).

In the oTS, differences in immune cell distribution were less pronounced (Fig. 2A). However, HPV-negative tumors still presented a greater percentage of fibroblasts (13.9% vs. 27.6%; **p = 0.008**) and macrophages (3.9% vs. 8.0%; **p = 0.02**). When examining cell state marker expression in the oTS, HPV-positive tumors presented increased fractions of B- cells expressing CD44, Ki67, HLA-DR, and CD79a, as well as plasma cells (CD79a^+^) and memory CD4^+^ *T*- cells (CD45RO^+^). In contrast, but similar to aTS, HPV-negative tumors were enriched with CD14^+^ macrophages and granulocytes, as well as with CD163^+^ M2-like macrophages (Fig. 2B–C, Suppl. Table S3B).

To identify specific cellular state enrichment within each cell type associated with the HPV phenotype, we compared the percentage of positive cell state markers within individual cell types in the adjacent (aTS) and outer tumor stroma (oTS) of the HPV-positive and HPV-negative tumors (Fig. 2D–E). Only biologically relevant and statistically robust combinations, defined as markers expressed in 10–80% of cells within a cell type and cell types comprising > 1% of all cells in an area, were included in the analysis (Suppl. Tables S2B-C). In the aTS of HPV-negative tumors, we observed significant enrichment of the *T*- cell exhaustion marker PD-1 in both CD8^+^ *T*- cells (mean positivity: 33.0% in HPV-negative vs. 16.7% in HPV-positive tumors; **p = 0.04**) and T_regs_ (22.5% vs. 8.2%; **p = 0.04**). Similarly, the terminal differentiation marker CD57 was more frequently expressed in these cell types in HPV-negative tumors (CD8^+^ *T*- cells: 7.4% vs. 2.0%; **p = 0.03**; T_regs_: 6.2% vs. 1.0%; **p = 0.003**). Conversely, in the aTS of HPV-positive tumors, we observed increased collagen IV positivity in myofibroblasts (45.2% vs. 19.7%; **p = 0.03**), increased proliferation (Ki67^+^) in macrophages (5.1% vs. 3.2%; **p = 0.02**), and elevated CD79a expression in plasma cells (57.3% vs. 30.6%; **p = 0.02**). Similar trends were observed in oTS, where HPV-negative tumors presented increased CD14 positivity in granulocytes (30.8% vs. 10.2%; **p = 0.03**) and increased PD-L1 expression in T_regs_ (15.1% vs. 4.4%; **p = 0.005**) (Fig. 2D–E, Suppl. Table S3C).

### HPV status is associated with distinct immune, stromal and tumor cell states within the tumor area of HNSCC

**Fig. 3:**
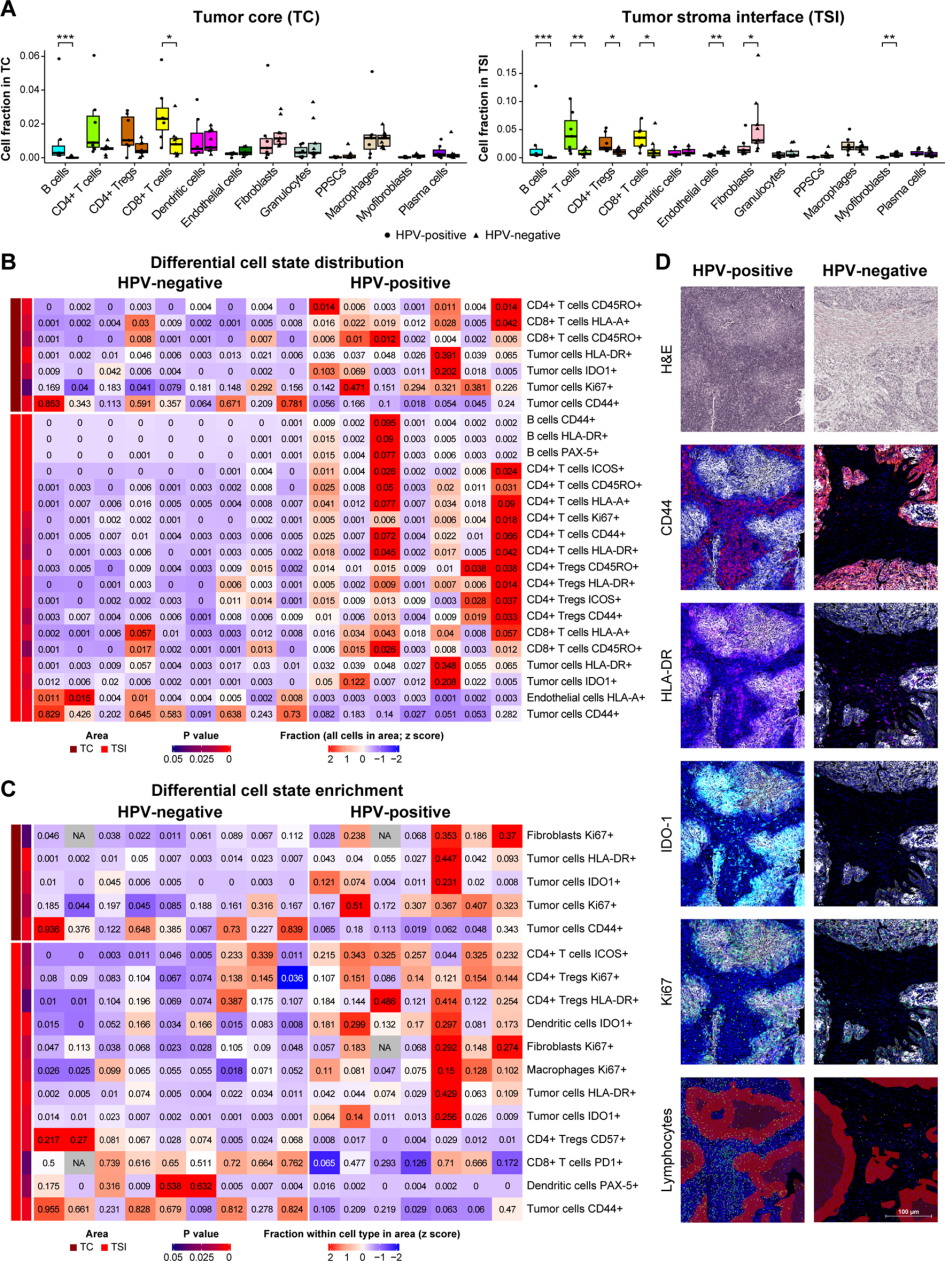
HPV status is associated with distinct immune, stromal and tumor cell states within the tumor area of HNSCC. (**A**) Box plots showing cell type fraction distributions in the tumor core (TC, left) and tumor stroma interface (TSI, right) for HPV-positive (*n* = 7) and HPV-negative (*n* = 9) tumors. (**B**) Heatmaps illustrating differentially distributed cell state fractions between HPV-positive (*n* = 7) and HPV-negative (*n* = 9) tumors in the TC and TSI. (**C**) Heatmap illustrating differential cell state enrichment between HPV-positive (*n* = 7) and HPV-negative (*n* = 9) tumors in the TC and TSI. (**D**) Representative H&E and mIF images of HPV-positive and HPV-negative tumors depicting variations in lymphocyte distribution and CD44, HLA-DR, and IDO1 expression. *p* values were calculated using the Mann‒Whitney U test. * = *p* < 0.05, ** = *p* < 0.01, *** = *p* < 0.001. PPSC; podoplanin-positive stromal cells

To investigate differences in the cellular composition and tumor cell states within the tumor area, we analyzed the fractions of various cell types and their associated activation markers. Within the tumor core (TC), greater percentages of B- cells (1.1% vs. 0.03%; **p = 0.0008**) and CD8^+^ *T*- cells (2.6% vs. 0.9%; **p = 0.02**) were observed in the HPV-positive group (Fig. 3A, Suppl. Table S3A). Similarly, in the tumor stroma interface (TSI), the tumor region bordering the tumor stroma presented significantly greater numbers of lymphocytes, including B- cells (mean cell fraction: 2.5% vs. 0.05%; **p = 0.0002**), CD4^+^ *T*- cells (4.5% vs. 0.9%; **p = 0.008**), and CD8^+^ *T*- cells (3.6% vs. 1.4%; **p = 0.03**). In contrast, HPV-negative tumors were enriched with stromal cells such as fibroblasts (5.6% vs. 2.0%; **p = 0.02**) and myofibroblasts (0.6% vs. 0.1%; **p = 0.002**). Interestingly, T_regs_ were more abundant in the TSI of HPV-positive tumors (2.6% vs. 1.0%; **p = 0.02**), whereas endothelial cells were more prevalent in HPV-negative tumors (1.0% vs. 0.4%; **p = 0.003**), despite neither cell type showing HPV-associated differences in the tumor–stroma (Fig. 3A, Suppl. Table S3A).

Analysis of cell state distributions in the TSI revealed a greater abundance of activated lymphocytes, marked by HLA-A and CD44 positivity, in HPV-positive tumors across B- cells, CD4^+^, CD8^+^, and T_regs_. Additionally, memory *T*- cells (CD45RO^+^), ICOS^+^ CD4^+^ and T_regs_ were enriched in HPV-positive tumors (Fig. 3B, Suppl. Table S3B). Tumor cells also exhibit distinct phenotypic states on the basis of their HPV status. HPV-positive tumors presented increased expression of immune-related proteins, including IDO1 (6.1% vs. 0.6%; **p = 0.01**) and HLA-DR (8.7% vs. 1.5%; **p = 0.005**), as well as a two-fold increase in proliferation (Ki67^+^ : 32.2% vs. 15.4%), as did memory *T*- cells (CD45RO^+^) and ICOS^+^ T_regs_. Conversely, CD44^+^ tumor cells remained more prevalent in the tumor core of the HPV-negative tumors (16.5% vs. 59.6%; **p = 0.005**) (Fig. 3B, Suppl. Table S3B).

Phenotypic enrichment analysis of lymphocytes revealed significantly greater ICOS expression in CD4^+^ *T*- cells in the TSI of HPV-positive tumors (24.9% vs. 7.1%; **p = 0.03**), whereas PD-1 expression in CD8^+^ *T*- cells (64.5% vs. 35.9%; **p = 0.04**) and CD57 expression in T_regs_ (9.3% vs. 1.0%; **p = 0.008**) were enriched in HPV-negative tumors. Additionally, we observed a greater proportion of proliferating fibroblasts (17.0% vs. 6.2%; **p = 0.02**) and macrophages (9.9% vs. 5.1%; **p = 0.01**) in HPV-positive tumors, as well as increased CD79a positivity in plasma cells (61.5% vs. 26.0%; **p = 0.01**) and IDO1 positivity in dendritic cells (19.1% vs. 6.0%; **p = 0.003**). Among tumor cells, IDO1 (7.4% vs. 0.7%; **p = 0.008**) and HLA-DR (11.3% vs. 1.8%; **p = 0.003**) were significantly elevated in HPV-positive tumors, whereas CD44 was enriched in HPV-negative tumors (16.5% vs. 59.6%; **p = 0.005**) across both the TC and TSI (Fig. 3C–D, Suppl. Table S3C).

### HPV status modulates the cellular neighborhood composition and functional state

**Fig. 4:**
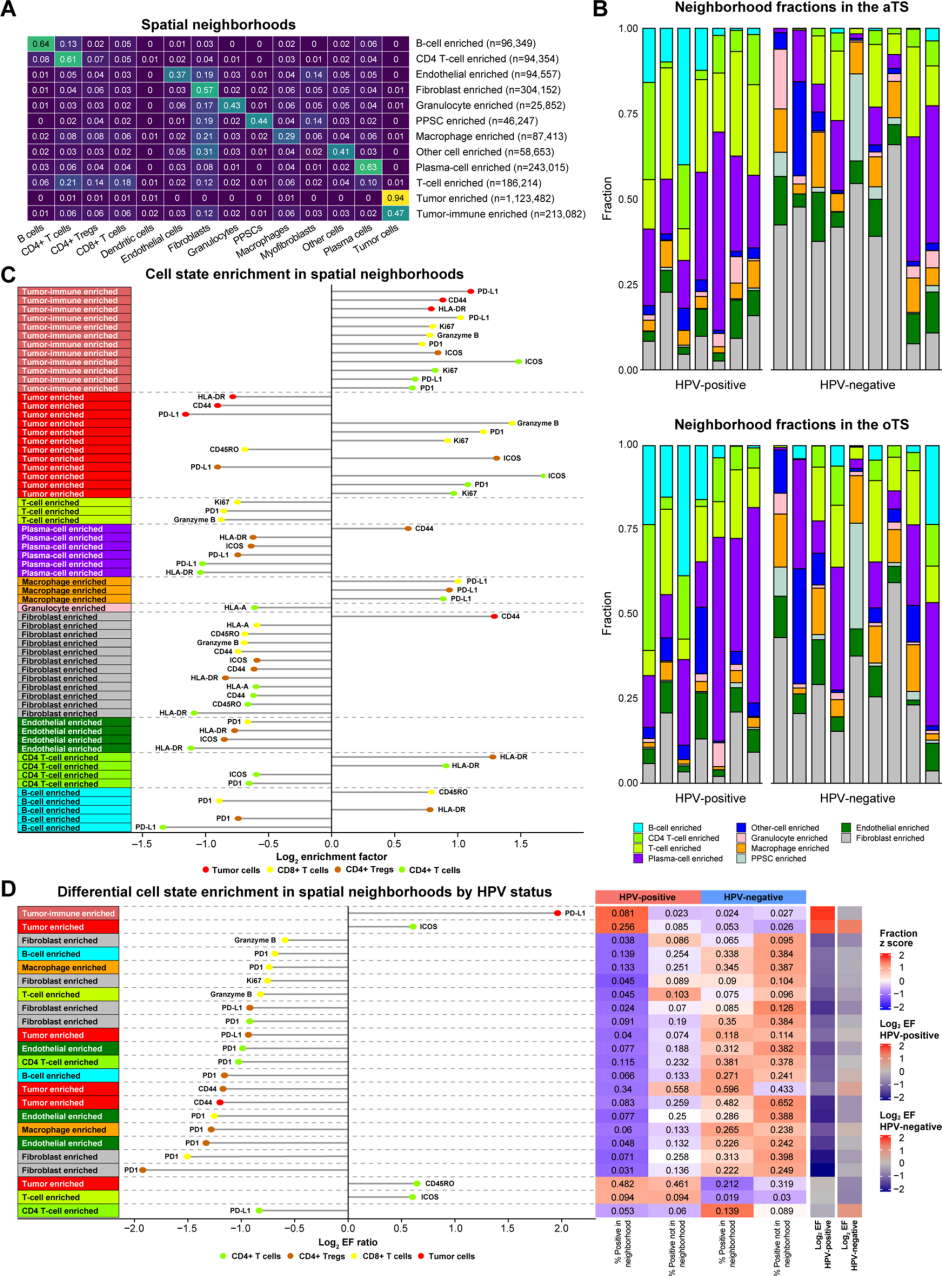
HPV status modulates the cellular neighborhood composition and functional state. (**A**) Heatmap showing the cellular neighborhood composition. (**B**) Bar plots depicting neighborhood fractions in the adjacent tumor stroma (aTS) and outer tumor stroma (oTS) for HPV-positive (*n* = 7) and HPV-negative (*n* = 9) tumors. (**C**) Dot chart depicting the differentially enriched (enriched factor > 1.5 or < 0.67, adjusted *p* value < 0.05) cell states within tumor cells and T cells in different cellular neighborhoods. (**D**) Dot chart depicting the differentially enriched (enrichment factor > 1.5 or < 0.67, adjusted *p* value < 0.05) ratio between HPV-positive (*n* = 7) and HPV-negative (*n* = 9) tumors, demonstrating differential tumor cell and T-cell state enrichment by HPV status (left), as well as the neighborhood and non-neighborhood cell state and log2 enrichment factor (right). *p* values were computed using Fisher’s exact test and adjusted for multiple corrections via the Benjamini–Hochberg procedure. PPSC; podoplanin-positive stromal cells

To further investigate the impact of HPV status on the micro-organization and activation states of cells, we employed a spatial analysis approach that captures co-regulated or co-functioning cellular structures. Cellular neighborhoods are defined as regions characterized by distinct local densities of various cell types [7]. For each cell, we identified its 10 nearest spatial neighbors to define a ‘spatial window’, capturing its immediate microenvironment (Suppl. Figure S2A). Clustering these spatial windows (k = 4–15), we selected k = 12 based on model fit, as assessed by the within sum of squares (WSS) and the Davies-Bouldin index (Suppl. Figure S2B), and biological interpretability. The resulting 12 spatial neighborhoods included 2 tumor-enriched regions (one composed of 94% tumor cells and another containing a mix of tumor, stromal, and immune cells, representing tumor-TME interactions), 4 single-cell dominant regions (enriched for B cells, CD4 *T*- cells, plasma cells and fibroblasts), and 6 mixed-cell neighborhoods enriched for endothelial, PPSC, stromal, or immune cells (*T*- cells, macrophages and granulocytes) (Fig. 4A).

HPV-positive and HPV-negative tumors presented distinct spatial neighborhood compositions within stromal compartments. HPV-positive tumors were enriched in B- cell, CD4 *T*- cell and *T*- cell enriched neighborhoods in the adjacent stroma, whereas HPV-negative tumors presented higher proportions of macrophage- and fibroblast-enriched neighborhoods across both adjacent and outer stromal regions (Fig. 4B, Suppl. Figure S2C; Suppl. Table S4A).

Analysis of cell state distributions within relevant neighborhood-cell type combinations (Suppl. Table S2D) and cell- marker combinations (Suppl. Table S2B) revealed that the most differentially represented cell types and states (absolute cell state fraction ratios between HPV-positive and HPV-negative tumors greater than 1.5 and *p* < 0.05) in HPV-positive tumors were enriched for activated *T*- cell (ICOS^+^, CD44^+^, HLA-A^+^ and CD45RO^+^) and CD44^+^ B- cells across B- cell, CD4 *T*- cell, endothelial, fibroblast, plasma and tumor-immune enriched neighborhoods. In contrast, HPV-negative tumors were characterized by increased fibroblasts (in plasma and tumor-immune enriched neighborhoods), macrophages (in endothelial, fibroblast, granulocyte, PPSC, and other enriched neighborhoods), and PD-L1^+^ CD4 *T*- cells (in CD4 *T*- cell–enriched neighborhoods) (Fig. S2D; Suppl. Table S4B).

To investigate how spatial micro-organization influences cellular function, we analyzed the enrichment of specific cell states across distinct spatial neighborhoods. Protein expression significance was quantified using an enrichment factor (EF), defined as the ratio of the protein-positive cell fraction within a given neighborhood to that in all other neighborhoods (EF > 1.5 or < 0.67, adjusted *p* < 0.05 by Fisher’s exact test). The top significantly enriched cell state was CD21^+^ B- cells within the B- cell enriched neighborhood (EF = 3.2, **p < 0.0001**; Suppl. Table S4C), which is consistent with germinal center formation and highlights the reciprocal relationship between spatial organization and cellular function. Focusing on *T*- cell and tumor cell states within different neighborhoods, in the Fibroblast-Enriched neighborhood, *T*- cells presented downregulation of activation markers, including CD44, CD45RO, HLA-A, ICOS, and Granzyme B, suggesting that fibroblasts play an immunosuppressive role. In contrast, within the Tumor-Immune enriched neighborhood (comprising ~50% tumor and ~50% stromal/immune cells), CD8^+^ *T*- cells were enriched for Granzyme B and PD-1 whereas CD4^+^ *T*- cells in this neighborhood were enriched for ICOS and PD-1. In the Tumor-enriched neighborhood, CD8^+^ *T*- cells presented reduced expression of CD45RO. Tumor cells in the Tumor-Immune enriched neighborhood expressed higher levels of PD-L1, HLA-DR, and CD44, which were notably downregulated in the Tumor-enriched neighborhood (Fig. 4C, Suppl. Table S4C).

Distinct immune neighborhoods are associated with specific T-cell states. In the macrophage-enriched neighborhood, T cells presented elevated PD-L1 expression, which is indicative of an immunosuppressive microenvironment. Conversely, the B Cell- enriched neighborhood supported T-cell activation, with CD8^+^ *T*- cells exhibiting increased CD45RO and reduced PD-1, and CD4^+^ *T*- cells showing decreased PD-L1. *T*- cells in the *T*- cell and CD4 *T*- cell enriched neighborhoods presented lower levels of PD-1, ICOS, and Granzyme B, which was consistent with the development of naive or memory-like phenotypes (Fig. 4C, Suppl. Table S4C).

To assess the impact of HPV status on cellular micro-organization and function, we compared cell state enrichment across spatial neighborhoods between HPV-positive and HPV-negative tumors (Fig. 4D, Suppl. Table S4D). In HPV-positive tumors, PD-L1^+^ tumor cells were enriched at the tumor-immune interface, and ICOS^+^ CD4 *T*- cells were enriched in the tumor core, suggesting enhanced tumor-reactive T-cell activity. *T*- cells in HPV-positive tumors also presented reduced PD-1 expression across multiple neighborhoods, indicating a less exhausted phenotype. In contrast, in addition to higher PD-1 positivity within *T*- cells, HPV-negative tumors enriched with PD-L1^+^ CD4 *T*- cells in CD4 *T*- cell enriched neighborhoods, which was consistent with more immunosuppressive microenvironments (Fig. 4D, Suppl. Table S4D).

### Tertiary lymphoid structures differ by HPV status in composition, functional state and tumor proximity

**Fig. 5:**
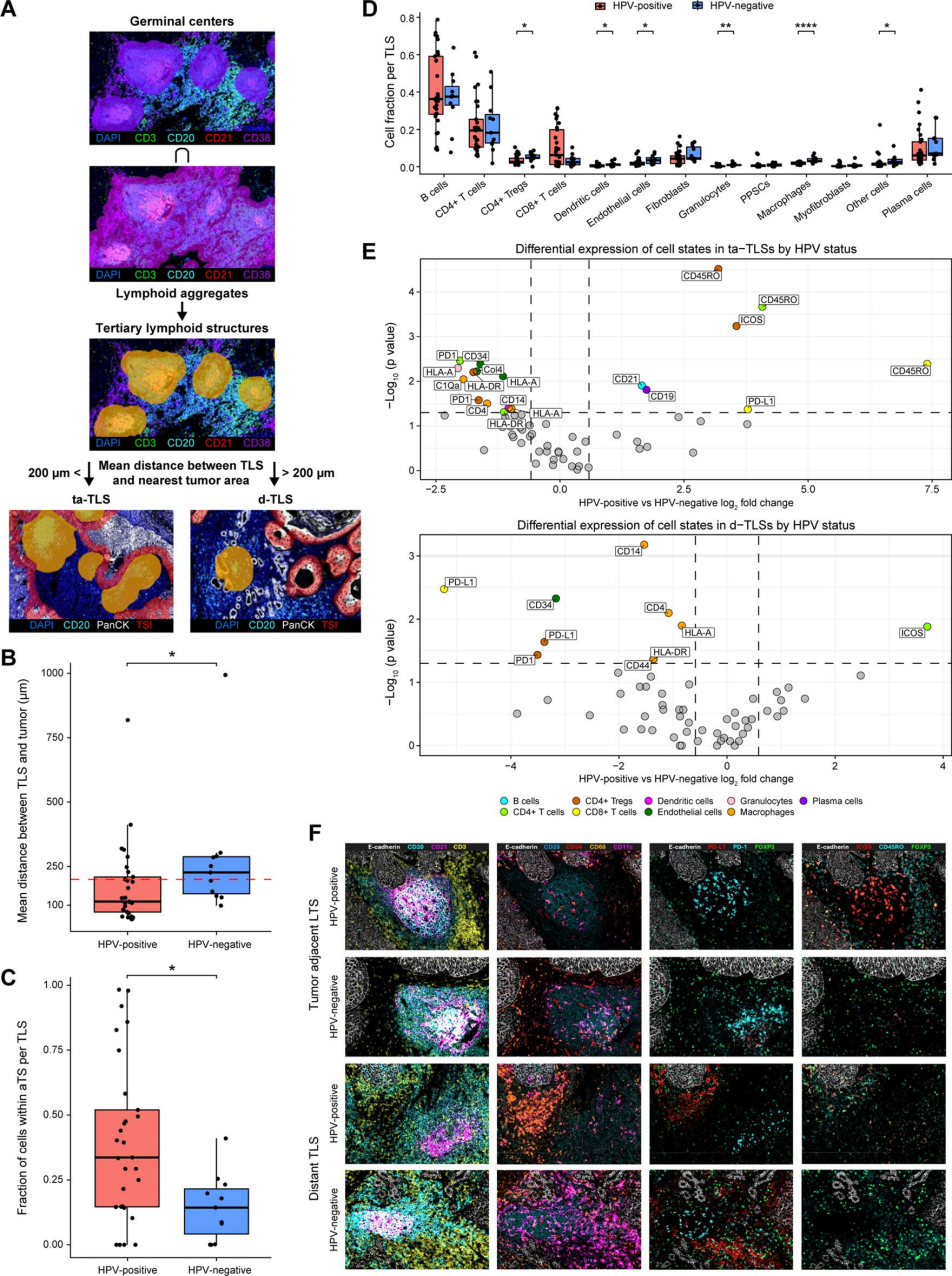
Tertiary lymphoid structures differ by HPV status in composition, functional state and tumor proximity. (**A**) Schematic representation of the TLS detection and segmentation model, including identification of tumor-adjacent (ta-TLS, mean distance < 200 µm from the nearest tumor area; *n* = 26) and distant (d-TLS, mean distance > 200 µm from the nearest tumor area; *n* = 14) TLSs. (**B**) Box plot showing the mean distance from TLSs to the nearest tumor area in HPV–positive (*n* = 7) and HPV-negative (*n* = 9) tumors. (**C**) Box plot demonstrating the fraction of TLS cells within the adjacent tumor stroma (aTS) in HPV–positive (*n* = 7) and HPV-negative (*n* = 9) tumors. (**D**) Box plots comparing differential cell fractions per TLS in HPV–positive (*n* = 7) and HPV-negative (*n* = 9) tumors. (**E**) Volcano plots demonstrating differential expression of cell states in ta-TLSs (top; *n* = 21 in HPV- positive and *n* = 5 in HPV-negative tumors) and d-TLSs (bottom; *n* = 8 in HPV- positive and *n* = 6 in HPV-negative tumors) by HPV status. The color of the dots represents the cell type, and the label represents phenotypic protein positivity. (**F**) Representative images of ta-TLSs and d-TLSs in HPV-positive and -negative tumors, showing distinct cellular compositions and activation states. *p* values were calculated using the mann‒Whitney U test. * = *p* < 0.05, ** = *p* < 0.01, *** = *p* < 0.001. PPSC; podoplanin-positive stromal cells

Next, we developed an automated TLS detection and segmentation model to identify mature TLSs and assess their spatial and compositional features. Germinal centers were defined as dense clusters of CD21^+^ B- cells and dendritic cells expanded by 100 µm to include the surrounding stroma and intersected with lymphoid aggregates identified by high densities of B- cells, CD4 *T*- cells, dendritic cells, and plasma cells (Fig. 5A). TLSs were detected in 4/7 HPV-positive and 4/9 HPV-negative tumors (Figure S3A), with HPV-positive tumors showing a trend toward a greater number of TLSs (HPV-positive *n* = 29; HPV-negative *n* = 11) per TLS-positive tumor (mean 7.25 vs. 2.75; **p = 0.11**; Figure S3B), whereas TLS size, estimated by cell count, did not differ significantly between groups (Suppl. Figure S3C). However, TLSs in HPV-positive tumors were significantly closer to tumor regions (mean distance 173 µm vs. 279 µm; **p = 0.03;** Fig. 5B,), with 37.9% located within 100 µm of tumor tissue compared with 9.1% in HPV-negative tumors (**p = 0.12**). Additionally, a greater proportion of cells in HPV-positive TLSs were within the adjacent tumor stroma (aTS) (mean 39.7% vs. 14.4%; **p = 0.02;** Fig. 5C), and we observed a trend toward a greater proportion of TLSs within the overall tumor stroma (17.9% in HPV-positive tumors vs. 6.4% in HPV-negative tumors) and aTS specifically (12.6% vs. 2.4%; Suppl. Fig. S3D).

Compared with those in HPV-negative TLSs, TLSs in HPV-positive tumors presented lower proportions of macrophages (1.8% vs. 3.6%; **p < 0.0001),** granulocytes (0.4% vs. 1.0%; **p < 0.002**), dendritic cells (0.6% vs. 1.4%; **p = 0.03**), CD4 Tregs (3.6% vs. 5.1**%**; **p = 0.04**) and endothelial cells (2.3% vs. 3.2%, **p = 0.05**) (Fig. 5D and Suppl. Table S5A). A comparison of the cell state distribution within TLSs revealed that HPV-negative TLSs were enriched in activated myeloid cells and immunosuppressive populations, including PD-1^+^ and PD-L1^+^ CD4 Tregs and CD4 *T*- cells. In contrast, HPV-positive TLSs were enriched for ICOS^+^ CD4 *T*- cells and Tregs, CD45RO^+^ CD8 *T*- cells, CD44^+^ and CD21^+^ B- cells, and CD19^+^ plasma cells (Suppl. Fig. S3E and Suppl. Table S5D), indicating a more active, immune-supportive microenvironment.

Given the emerging role of tumor-adjacent TLSs [20], we next compared the TLS composition on the basis of their proximity to tumor tissue, classifying TLSs as either tumor-adjacent (ta-TLSs, mean distance ≤200 µm from the nearest tumor area; *n* = 26) or distant (d-TLSs, mean distance > 200 µm from the nearest tumor area; *n* = 14) (Fig. 5A). The only significant difference in cell type distribution between ta-TLSs and d-TLSs was the greater proportion of PPSCs within the ta-TLSs (1.2% vs. 0.04%; **p = 0.02**; Suppl. Table S5C). However, cell state distribution analysis revealed increased proportions of activated B- cells (CD79a^+^/CD21^+^), plasma cells (CD44^+^/CD79a^+^), and PPSCs (CD44^+^/HLA-A^+^) in ta-TLSs as well as ICOS^+^ Tregs, whereas d-TLSs presented increased proportions of CD4^+^ macrophages (Suppl. Figure S3F and Suppl. Table S5D).

Comparing ta-TLSs by HPV status revealed that HPV-positive tumors had increased numbers of CD21^+^ B- cells, CD19^+^ plasma cells, ICOS^+^ CD4 Tregs, and memory *T*- cells (CD45RO^+^) across the CD4, CD8, and T_reg_ subsets (Fig. 5E‒F and Suppl. Table S5E‒F), indicating enhanced immunological activation and maturation within these TLSs. In contrast, HPV-negative ta-TLSs were enriched for endothelial cells, granulocytes, C1Qa^+^ macrophages, and PD-1^+^ CD4 *T*- cells and Tregs (Fig. 5E–F and Suppl. Table S5E-F), indicating a more suppressive phenotype. Similar trends were observed in d-TLSs, where HPV-positive TLSs had more ICOS^+^ CD4 *T*- cells, whereas HPV-negative TLSs were enriched in macrophages, PD-1^+^/PD-L1^+^ CD4 Tregs, and PD-L1^+^ CD8 *T*- cells (Fig. 5E–F and Suppl. Table S5E-F).

### Spatially resolved cell‒cell interactions and principal component analysis reveal distinct HPV-associated immune profiles

Spatially resolved protein data enable analysis beyond cellular composition, allowing us to examine cell-cell interactions, activation states and their association with HPV status. We computed a set of spatial features capturing the frequency of interactions between neighboring cells, incorporating phenotypic states. Interaction frequency was quantified as the fraction of cells with at least one neighboring cell within 25 µm, based on centroid-to-centroid distances measured within a given area. After filtration of highly correlated features (Pearson coefficient threshold < 0.8), 570 features were analyzed across all tumor regions to identify interaction patterns enriched in HPV-positive vs. HPV-negative tumors.

**Fig. 6:**
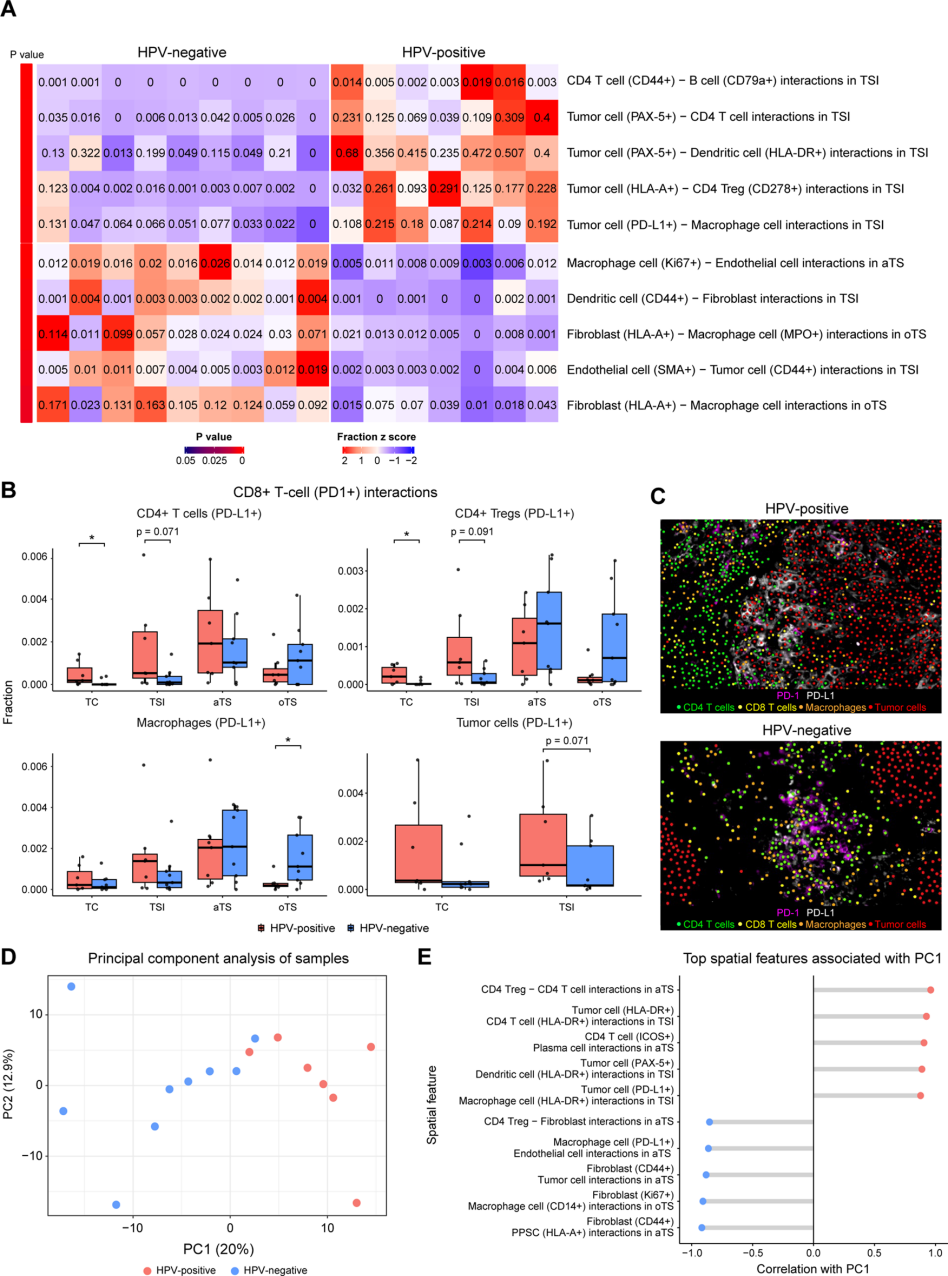
Spatially resolved cell interactions and principal component analysis revealed distinct HPV-associated immune profiles. (**A**) Heatmap showing the top 5 differentially distributed cell‒cell interactions, defined as the fraction of cells with at least one neighboring cell within 25 µm, based on centroid-to-centroid distances, within a given area of HPV-positive (*n* = 7) and HPV-negative (*n* = 9) tumors. (**B**) Box plots demonstrating differential interaction frequencies between PD-1^+^ CD8^+^ T cells and PD-L1^+^ cells in TC (tumor core), TSI (tumor-stroma interface), aTS (adjacent tumor stroma), oTS (outer tumor stroma) areas of HPV-positive (*n* = 7) and HPV-negative (*n* = 9) tumors. (**C**) Representative images of PD-1 and PD-L1 interactions. (**D**) Scatter plot depicting the distribution of HPV-positive (*n* = 7) and HPV-negative (*n* = 9) tumors in relation to the first two primary components. Principal component analysis was conducted on the 500 most variable normalized spatial features, following filtration of lowly expressed and highly correlated features. (**E**) Dot chart of the top 5 spatial features that are strongly and inversely correlated with PC1. *p* values were calculated using the Mann‒Whitney U test. * = *p* < 0.05

In HPV-positive tumors, the most enriched interactions included those between activated (CD44^+^) CD4 *T*- cells and CD79^+^ B- cells within the tumor–stroma interface (mean frequency of interaction of 0.88% HPV-positive vs. 0.04% HPV-negative; **p = 0.0002**), as well as interactions between tumor cells and immune populations, including CD4 T-cells, CD4 T_regs_, dendritic cells, and macrophages (all **p < 0.005**). In contrast, HPV-negative tumors exhibited increased interactions between stromal and immune or tumor cells, particularly between macrophages and fibroblasts or endothelial cells (**p < 0.01**), and between tumor cells and endothelial cells (0.28% HPV-positive vs. 0.88% HPV-negative; **p = 0.003**; Fig. 6A, Suppl. Table S6A).

We further investigated immune checkpoint interactions by analyzing the spatial proximity between PD-1^+^ *T*- cells and PD-L1^+^ cells. Across all tumor regions, the interaction frequency, calculated as detailed above, between PD-1^+^ CD8 *T*- cells and PD-L1^+^ cells was greater in HPV-positive tumors. In both the TC and TSI of HPV-positive tumors, we observed a greater interaction frequency between PD-1^+^ CD8 *T*- cells and PD-L1^+^ CD4 T-cells (mean frequency of 0.047% vs 0.008%; **p = 0.03** in the TC, 0.17% vs. 0.03%; **p = 0.07** in the TSI) and PD-L1^+^ CD4 Tregs (mean frequency of 0.025% vs 0.004%; **p = 0.03** in the TC, 0.09% vs. 0.02%; **p = 0.09** in the TSI). We also observed a trend toward a greater frequency of PD-1^+^ CD8 *T*- cell and PD-L1^+^ tumor cell interactions within the TSI of HPV-positive tumors (mean frequency of 0.2% vs 0.09%; **p = 0.07**). In contrast, HPV-negative tumors presented a greater prevalence of PD-1^+^ *T*- cell interactions with PD-L1^+^ macrophages in the oTS (mean interaction frequency of 0.03% in HPV-positive vs. 0.16% in HPV-negative tumors; **p = 0.03**), suggesting distinct mechanisms of immune suppression based on HPV status (Fig. 6B–C and Suppl. Table S6B). Similar trends were observed for PD-1^+^ CD4 *T*- cell interactions with PD-L1^+^ cells (Figure S5A and Suppl. Table S6B). Interaction analysis within the TLS microdomains showed greater interactions between PD-1^+^ CD4 *T*- cell with PD-L1^+^ macrophages and PD-L1^+^ CD4 cells in HPV- negative tumors similar to the oTS interaction scores (Figure S5B and Suppl. Table S6C).

Finally, to identify the principal determinants of spatial variability within the cohort, we integrated all computed spatial features characterizing cellular and phenotypic state distributions, as well as cell–cell interactions and proximities within defined tissue regions and neighborhoods (Suppl. Fig. S4). Following the exclusion of lowly expressed and highly correlated features (Pearson correlation coefficient threshold < 0.9), a total of 2,204 spatial features were retained for downstream analysis. Principal component analysis (PCA) of the 500 most variable spatial features revealed that the first principal component (PC1), accounting for 20.04% of the total variance, significantly distinguished the HPV-positive samples from the HPV-negative samples (*p* = 0.0003; Fig. 6D, Suppl. Fig. S5B). The spatial features most strongly associated with PC1 (Pearson correlation coefficient > 0.87) were enriched in HPV-positive tumors and reflected elevated lymphocyte infiltration into both the tumor and adjacent stromal compartments. These included interactions between CD4^+^ *T*- cells and CD4^+^ Tregs, as well as plasma cells within the aTS. Additionally, tumor cell interactions with MHC class II–expressing antigen-presenting cells, such as dendritic cells and macrophages, are prominent at the tumor–stroma interface. Conversely, spatial features that were negatively correlated with PC1 (Pearson correlation coefficient < −0.85) and thus enriched in HPV-negative tumors were characterized by increased interactions between stromal components (fibroblasts, endothelial cells, and PPSCs) and immunosuppressive cell types, including PD-L1^+^ macrophages and CD4^+^ Tregs, within the tumor stroma (Fig. 6E; Suppl. Table S6D).

## Discussion

The tumor microenvironment in HNSCC represents a complex and dynamic ecosystem [5] in which immune and stromal components interact with malignant cells to shape disease progression, therapeutic response, resistance and survival [24, 25]. HPV-positive HNSCC tumors are characterized by a highly inflamed TME [12, 22] with abundant tumor-infiltrating lymphocytes (TILs), a feature that is consistently linked to improved clinical outcomes [4, 26]. Notably, not only the abundance of TILs but also their spatial distribution, particularly the proximity and interaction of CD8^+^ *T*- cells with tumor cells, distinguishes responders from non-responders to therapy [27, 28]. In this study, we employed a high-plex immunofluorescence approach combined with deep-learning-based image analysis to dissect the spatial architecture and cellular composition of the TME in HPV-positive and HPV-negative HNSCC tumors. This computational framework enabled high-accuracy cell classification, revealing that the primary axis of biological variability between samples was driven by HPV status, with HPV-positive tumors defined by immune infiltration and HPV-negative tumors defined by stromal fibrosis. This finding underscores that our spatial profiling successfully identified distinct immunological and stromal landscapes, providing a robust foundation for our detailed observations.

Our findings confirm and expand upon the established paradigm of HPV-positive HNSCC as an immunologically “hot” tumor [12, 22]. HPV-positive tumors exhibit a highly inflamed TME enriched with TILs, including CD8^+^, CD4^+^, Treg, and B- cells [12, 29, 30], particularly in tumor-adjacent regions [12]. This spatial arrangement of immune cells, concentrated at the invasive front, has been previously associated with a favorable prognosis and is crucial for effective anti-tumor immunity [31]. In contrast, HPV-negative tumors present with a fibrotic and immunosuppressive TME, characterized by an enrichment of cancer-associated fibroblasts (CAFs), myofibroblasts, and M2- like macrophages [12, 32, 33]. These findings are consistent with prior single-cell studies that reported differences in lymphoid and myeloid cell states between HPV-positive and HPV-negative HNSCC [12, 22, 34, 35], while adding critical spatial context to these observations. Notably, not only was the abundance of lymphocytes in HPV-positive biopsies greater, but they also displayed an activated phenotype, with increased expression of markers such as CD44, CD45RO, HLA-A and ICOS. These findings suggest that immune infiltration in these tumors represents an active and antigen-driven adaptive immune response. Additionally, HPV-positive tumor cells presented increased expression of immune-related proteins, including IDO1 and HLA-DR, which is consistent with the viral origin of these tumors and previous reports of enriched interferon signaling [36–38]. In contrast, HPV-negative tumor cells presented elevated CD44 expression, which is consistent with prior transcriptomic data [14], suggesting a more transformed and stem-like phenotype [39, 40] that may contribute to their reduced clinical response and resistance to therapy.

To further delineate the distinct immune landscapes of HPV-positive and HPV-negative HNSCC, we analyzed their cellular states, spatial neighborhoods, and cell-cell interactions. In HPV-negative tumors, T cells within the adjacent stroma exhibit elevated expression of exhaustion markers such as PD-1 and CD57, indicative of functional suppression and senescence [41]. These tumors also show enrichment of immunosuppressive macrophages (CD163^+^) and granulocytes, along with increased interactions between PD-1^+^ CD8^+^ T cells and PD-L1^+^ macrophages in the outer tumor stroma, collectively contributing to a “cold” immune phenotype and forming a functional barrier to immune infiltration [42]. Additionally, HPV-negative tumors present a greater abundance of fibroblasts and fibroblast-enriched neighborhoods, where all T cells display reduced expression of activation markers such as CD44, HLA-A, CD45RO, ICOS, and Granzyme B. These findings suggest that fibroblasts may also contribute to a physical barrier that limits anti-tumor activity, reinforcing the “cold” immunosuppressive nature of these tumors [43, 44]. In contrast, HPV-positive tumors present an immune-supportive microenvironment with abundant B- and T-cell–enriched niches. Although fibroblasts were less abundant in HPV-positive tumors, those present presented elevated Col IV and CD44 expression. Spatial transcriptomics has revealed a fibroblast subset enriched in HPV-positive tumors and linked to an improved immunotherapy response [22]. Tumor cells within these immune-enriched neighborhoods demonstrated PD-L1 positivity, and within the tumor area, we observed frequent interactions between PD-1^+^ T cells and PD-L1^+^ cells, as well as ICOS^+^ CD4^+^ T cells, indicating a more active, tumor-reactive phenotype [45]. T cells within tumor-enriched neighborhoods presented increased expression of effector and exhaustion markers such as PD-1, Granzyme B, and ICOS, whereas those in stromal-enriched neighborhoods presented reduced activation marker expression. For example, in the tumor-immune-enriched neighborhood, CD8^+^ T cells were enriched for Granzyme B, PD-1, and Ki-67, indicative of strong anti-tumor activity and terminal exhaustion [46] while retaining their proliferative capacity [38]. In contrast, CD8^+^ T cells in the tumor-enriched neighborhood presented downregulation of CD45RO and Ki-67, suggesting a transition to a terminally differentiated effector state [47].

Neighborhood-specific modulation of CD8^+^ T-cell states was also evident in other immune contexts: in B cell- enriched neighborhoods, CD8^+^ T cells presented elevated CD45RO and reduced PD-1 expression, which was consistent with a more activated memory phenotype; in macrophage-enriched neighborhoods, CD8^+^ T cells were enriched for PD-L1 expression, reflecting a more inhibitory milieu. Notably, PD-L1 positivity in these contexts may be influenced by signal spillover from adjacent PD-L1^+^ macrophages, warranting cautious interpretation of this marker in densely interactive regions.

This study further highlights the significant role of TLSs in shaping the antitumor immune response in an HPV-dependent manner. Prior studies have reported a greater frequency of intra- and peritumoral TLSs in HPV-positive HNSCC patients and linked mature TLSs with improved survival [18]. Additionally, the closer proximity of TLSs to tumor cells has been associated with better response to immune checkpoint inhibitors [20]. Our analysis revealed that HPV-positive tumors harbored more mature, tumor-proximal TLSs enriched with ICOS^+^ CD4^+^ *T*- cells and CD45RO^+^ *T*- cells, as well as CD21^+^ B- cells, supporting the presence of mature TLSs and active immune priming [48–50]. The proximity of these immune-organizing hubs to malignant cells provides a more efficient platform for antigen presentation and T-cell priming, fostering a robust local immune response. In contrast, HPV-negative tumors presented more distant and less mature TLSs, lacked the immune-activating features observed in their HPV-positive counterparts, and were enriched with immune-inhibiting cells such as PD-1^+^ and PD-L1 ^+^ *T*- cells and macrophages. These spatial differences in TLS architecture and composition may serve as predictive biomarkers for immunotherapeutic efficacy and likely contribute to the enhanced responsiveness to immune checkpoint inhibitors observed in HPV-positive HNSCC.

This study has limitations that should be considered when interpreting the findings. While the sample size is relatively modest, the consistency of observed patterns across multiple analyses supports the robustness of our findings. These results provide a strong foundation for future validation in larger, independent clinical cohorts. Second, the absence of clinical outcome data precludes direct correlations between spatial or phenotypic features and treatment response, and future integration of clinical endpoints will be essential to fully translate these observations. Finally, while the deep learning-based analytical pipeline demonstrated high accuracy and concordance with expert annotations, complex tissue contexts, such as overlapping or engulfed cells, may still pose challenges for perfect cell boundary delineation and classification.

## Conclusion

In summary, our study highlights the impact of HPV status on the immune landscape, stromal composition, and spatial organization of the TME in HNSCC. The enhanced immune activation, mature TLSs, and antigen-presenting tumor cells in HPV-positive tumors likely underlie their improved clinical outcomes and responsiveness to immune checkpoint blockade. Conversely, the T-cell exhaustion and myeloid suppression observed in HPV-negative tumors may necessitate combination therapy that overcomes these specific resistance mechanisms. For example, combining checkpoint blockade with therapies that target immunosuppressive macrophages, or stromal cells could be a more effective strategy for HPV-negative patients. These hypothesis-generating findings underscore the importance of spatially resolved, high-dimensional profiling in revealing actionable features of the TME and pave the way for more personalized and effective therapeutic strategies in HNSCC. The observed associations between TLS proximity and cellular composition highlight their potential as candidate biomarkers, which merit further investigation and validation in larger, independent cohorts before clinical utility can be established.

## Electronic supplementary material

Below is the link to the electronic supplementary material.

Supplementary Material 1

Supplementary Material 2

Supplementary Material 3

Supplementary Material 4

Supplementary Material 5

Supplementary Material 6

## Data Availability

Data generated or analyzed during this study are included in this published article and its supplementary information files. The code used for analysis is not publicly available due to its proprietary nature. Image files are available upon reasonable request.

## Abbreviations

HNSCC: Head and Neck Squamous Cell Carcinoma
HPV: Human Papillomavirus
TME: Tumor Microenvironment
TLS: Tertiary Lymphoid Structure
mIF: Multiplex Immunofluorescence
FFPE: Formalin-Fixed, Paraffin-Embedded
CNN: Convolutional Neural Network
WSI: Whole Slide Image
ROI: Region of Interest
TC: Tumor Core
TSI: Tumor-Stroma Interface
aTS: Adjacent Tumor Stroma
oTS: Outer Tumor Stroma
ta-TLS: Tumor-Adjacent Tertiary Lymphoid Structure
d-TLS: Distant Tertiary Lymphoid Structure
PCA: Principal Component Analysis
EF: Enrichment Factor
PD-1: Programmed Cell Death Protein 1
PD-L1: Programmed Death-Ligand 1
ICOS: Inducible T-cell CO-Stimulator
HLA-DR: Human Leukocyte Antigen – DR isotype
IDO1: Indoleamine 2,3-dioxygenase 1
CD44: Cluster of Differentiation 44
CD57: Cluster of Differentiation 57
CD45RO: Cluster of Differentiation 45RO
CD79a: Cluster of Differentiation 79a
PPSC: Podoplanin-Positive Stromal Cells
