# Supplementary material for: Spatial profiling of HPV-stratified head and neck squamous cell carcinoma reveals distinct immune niches and microenvironmental architectures
Source: J Transl Med. 2025 Nov 18;23:1304. doi: 10.1186/s12967-025-07280-x (PMC12625076; doi:10.1186/s12967-025-07280-x)
Supplement: Supplementary file 1 — Supplementary Material 1 [file 12967_2025_7280_MOESM1_ESM.pdf]

Spatial Profiling of HPV-Stratified Head and Neck Squamous Cell Carcinoma Reveals Distinct Immune Niches and Microenvironmental Architectures

Ettai Markovits, Dmytro Klymyshyn, Roni Froumine, Hailing Zong, Michael Mints, Sangeetha Mahadevan, Kenneth Bloom, Jamie Bates, Gareth J Thomas, Lauri Diehl, Oscar Puig, Abhishek Aggarwal

Table of contents

Supplementary Materials and Methods……………………………………………………………2

Fig. S1 ……………………………………………………………………………………………6

Fig. S2 ……………………………………………………………………………………………7

Fig. S3 ……………………………………………………………………………………………8

Fig. S4 ……………………………………………………………………………………………9

Fig. S5 ……………………………………………………………………………………………10

Supplementary Table 1……………………………………………………………………………11

List of Additional Supplementary Tables…………………………………………………………12

Supplementary References …………………………………………………………………………12

Supplementary Materials and Methods

Multiplex immunofluorescence using PhenoCycler-Fusion

Multiplex immunofluorescence (mIF) staining and whole-slide imaging were performed using the PhenoCycler-Fusion platform (Akoya Biosciences, USA) as previously described[1]. FFPE tissue sections (5 µm) were baked at 60°C for 12 hours, followed by standard deparaffinization and rehydration. Slides were rinsed with distilled water to remove residual ethanol prior to antigen retrieval. Epitope retrieval was performed by incubating slides in Tris-EDTA buffer (pH 9; #S2367, Dako) for 20 minutes in a pressure cooker. Slides were then equilibrated at room temperature for 30 minutes, rinsed, and stored in Hydration Buffer (#7000017, Akoya Biosciences) until staining. An antibody cocktail was prepared using optimized dilutions in a blocking buffer containing N, J, S, and G blockers (#7000017, Akoya Biosciences). Custom carrier-free antibodies were conjugated to PhenoCycler barcodes using the antibody conjugation kit. Slides were rinsed with Staining Buffer (#240198), fixed with 1.6% paraformaldehyde (PFA; #15710, Electron Microscopy Sciences) in Storage Solution (#232107), and incubated with the antibody cocktail for 3 hours at room temperature. For imaging, a flow cell (#240204) was assembled on each slide per manufacturer instructions, and slides were imaged using the PhenoCycler-Fusion system. Final image files (qptiff format) were visualized and analyzed using QuPath v0.5.1 (https://qupath.github.io/).

Antibody	Dilution	Clone	Catalog #	Barcode/Channel
HLA-A	1:200	EP1395Y	4450046	BX004-AF750
CD34	1:100	QBEND/10	4250057	BX025-ATTO550
CD4	1:200	EPR6855	4550112	BX003-Cy5
CD20	1:200	L26	4450018	BX007-AF750
CD14	1:500	EPR3653	4450047	BX037-ATTO550
CD68	1:100	KP1	4550113	BX015-Cy5
Vimentin	1:200	O91D3	4450050	BX022-AF750
CD8	1:200	C8/144B	4250012	BX026-ATTO550
CD11c	1:200	118/A5	4550114	BX024-Cy5
CD31	1:100	EP3095	4450017	BX001-AF750
E-Cadherin	1:300	4A2C7	4250021	BX014-ATTO550
CD45	1:300	D9M81	4550121	BX021-Cy5
SMA	1:200	1A4	4450049	BX013-AF750
CD45RO	1:200	UCHL1	4250023	BX017-ATTO550
CD3e	1:200	EP449E	4550119	BX045-Cy5
Pan-Cytokeratin	1:500	AE1/AE3	4450020	BX019-AF750
CD44	1:400	156-3C11	4450041	BX005-ATTO550
HLA-DR	1:200	EPR3692	4550118	BX033-Cy5
CD79a	1:200	D1X5C	4450078	BX090-AF750
GranzymeB	1:100	D6E9W	4250055	BX041-ATTO550
Collagen-IV	1:200	EPR209660	4550122	BX042-Cy5
C1Qa	1:50	EPR29804	Custom	BX070-AF750
Podoplanin	1:200	NC-08	4250004	BX023-ATTO550
FoxP3	1:100	236A/E7	4550071	BX031-AF647

LAG3	1:50	EPR20261	4550058	BX055-ATTO550
CD163	1:100	D6U1J	Custom	BX016-AF647
ICOS	1:200	D1K2T	4550117	BX054-ATTO550
IDO1	1:200	V1NC3IDO	4550123	BX027-Cy5
CD19	1:50	RM332	Custom	BX028-ATTO550
PAX-5	1:100	RM331	Custom	BX088-Cy5
Ki67	1:300	B56	4250019	BX047-ATTO550
CD21	1:300	EP3093	4450027	BX032-ATTO550
CD38	1:100	E7Z8C	4250080	BX089-ATTO550
PD-1	1:100	D4W2J	4550038	BX046-AF647
MPO	1:200	E1E7I	4250083	BX098-ATTO550
PD-L1	1:100	RM320	4550072	BX043-AF647
CD57	1:100	HNK-1	Custom	BX049-AF647

Cell Typing and Marker Quantification

Multiplex immunofluorescence (mIF) whole-slide images (WSIs) were analyzed using Nucleai's mIF analysis pipeline[2]. Regions of interest and image artifacts (e.g., out-of-focus areas, staining defects, tissue folds) were manually annotated. Cells were detected and segmented using a deep learning algorithm (NucleAI, Israel), utilizing a U-Net architecture with 8 million parameters[3]. This algorithm was trained on over 10,000 cell segmentation annotations from expert annotators across multiple mIF datasets. These datasets included a range of image resolutions and bit depths, specifically 8-bit and 16-bit, to ensure robust performance across diverse imaging conditions. To normalize staining intensities across slides, a stain normalization algorithm (NucleAI, Israel)[2] was applied, enhancing signal-to-noise ratio and standardizing channel intensities across the cohort. Marker positivity was inferred using a deep learning-based binary classifier[2], which outputs per-cell probabilities for each marker. Thresholds were optimized per channel by maximizing the F1 score and applied to convert probabilities into binary classifications.

Dynamic range thresholds were defined per channel: the lower bound was set to twice the peak of the cell intensity density plot, and the upper bound to the 90th percentile, constrained by the adjusted lower bound. Cell types were assigned based on exact matches to expected marker expression profiles. For unmatched cells, the five nearest neighbors in the marker probability feature space were used to assign the most frequent cell type. Marker positivity was validated using 78,320 single-channel annotations; cell typing was evaluated on 4,446 annotated cells.

H&E- Based Tissue Segmentation

A convolutional neural network (CNN) was trained on 190 expert-annotated regions of interest (ROIs) to segment tissue into four categories: tumor, tumor-stroma, necrosis, and other tissue types. Validation was performed on an independent set of 35 ROIs, with minor manual corrections applied to 12

images. This model classified regions into four primary categories: tumor, tumor-associated stroma, necrotic tissue, and “other” areas, which included benign epithelium, large blood vessels, and adipose tissue. Necrotic regions were excluded from downstream analyses. Tumor regions were further subdivided into the following compartments based on their spatial relationship to the tumor-stroma border:

- Tumor Core (TC): Tumor area located more than 60 µm away from the tumor-stroma interface.
- Tumor-Stroma Interface (TSI): Tumor area within 60 µm of the tumor-stroma border.
- Adjacent Tumor-Stroma (aTS): Tumor-stroma area within 60 µm of the tumor-stroma border.
- Outer Tumor-Stroma (oTS): Tumor-stroma area located more than 60 µm from the tumor-stroma border.

Multiplex immunofluorescence (mIF) images were registered to H&E images using a two-step alignment process. First, hematoxylin-only images were extracted via color deconvolution and aligned to the DAPI channel using rigid transformation based on normalized cross-correlation followed by optical flow-based correction to account for local deformations.

Cellular neighborhoods identification

Cellular neighborhoods were identified as previously described[4]. In short, a “spatial window” was defined around each detected cell by including its 10 nearest neighbors along with the cell of origin. Cell-type counts within each window were normalized using an inverse hyperbolic sine transformation (cofactor = 5) to emphasize diverse interactions over those dominated by a single cell type. A total of 300,000 cells were sampled, with balancing across samples and cell types. K-means clustering (k = 4–15) was applied to identify distinct cellular neighborhoods. The optimal number of clusters (k = 12) was selected based on goodness-of-fit metrics, including the Within-Cluster Sum of Squares and the Davies-Bouldin Index[5], as well as biological interpretability. Each cell was then assigned to a neighborhood based on its proximity to the centroid vector of mean neighbor compositions within each cluster.

Spatial features calculation

Spatial features were calculated to capture cell distributions, cellular states (cell type combined with marker positivity), and cell-cell interactions. These features included cell type and cell state fractions from all cells within a compartment (area, neighborhood or a TLS) and cell state enrichment within cells in a compartment. Cell-cell interactions and receptor-ligand interactions, such as PD-1/PD-L1, were quantified by calculating the fraction of cells with at least one neighboring cell within 25 µm, based on distances measured between cell centroids. Admissibility tables were used to define the feature space (**Suppl. Tables S2B-E**), allowing only biologically relevant cell-marker, with marker positivity

constrained to 10–80% within a cell type, Cell-compartment combinations were deemed admissible if the cell fraction within the area was higher than 1%. For rare or low-frequency markers, admissibility was defined by having a fraction above the average across all cells. All admissibility tables were manually reviewed and adjusted to ensure capturing of all biologically relevant spatial features. In instances where the denominator for a spatial feature was less than 30, the feature was assigned a value of "not applicable" (NA) for that specific sample, ensuring statistical robustness and reliability. For cell state enrichment within cells analysis, a protein positivity threshold of 5% in at least one of the HPV groups was used, to exclude low abundance cell states. Cell-cell interaction features were excluded if their prevalence was below 0.1% of the area in more than 90% of samples or if they contained more than 25% missing values. To address feature redundancy, we implemented a systematic approach to remove highly correlated features. Features were ranked based on variance, and starting with the most variable feature, each subsequent feature was iteratively added only if its Pearson correlation coefficient with previously selected features was below a threshold.

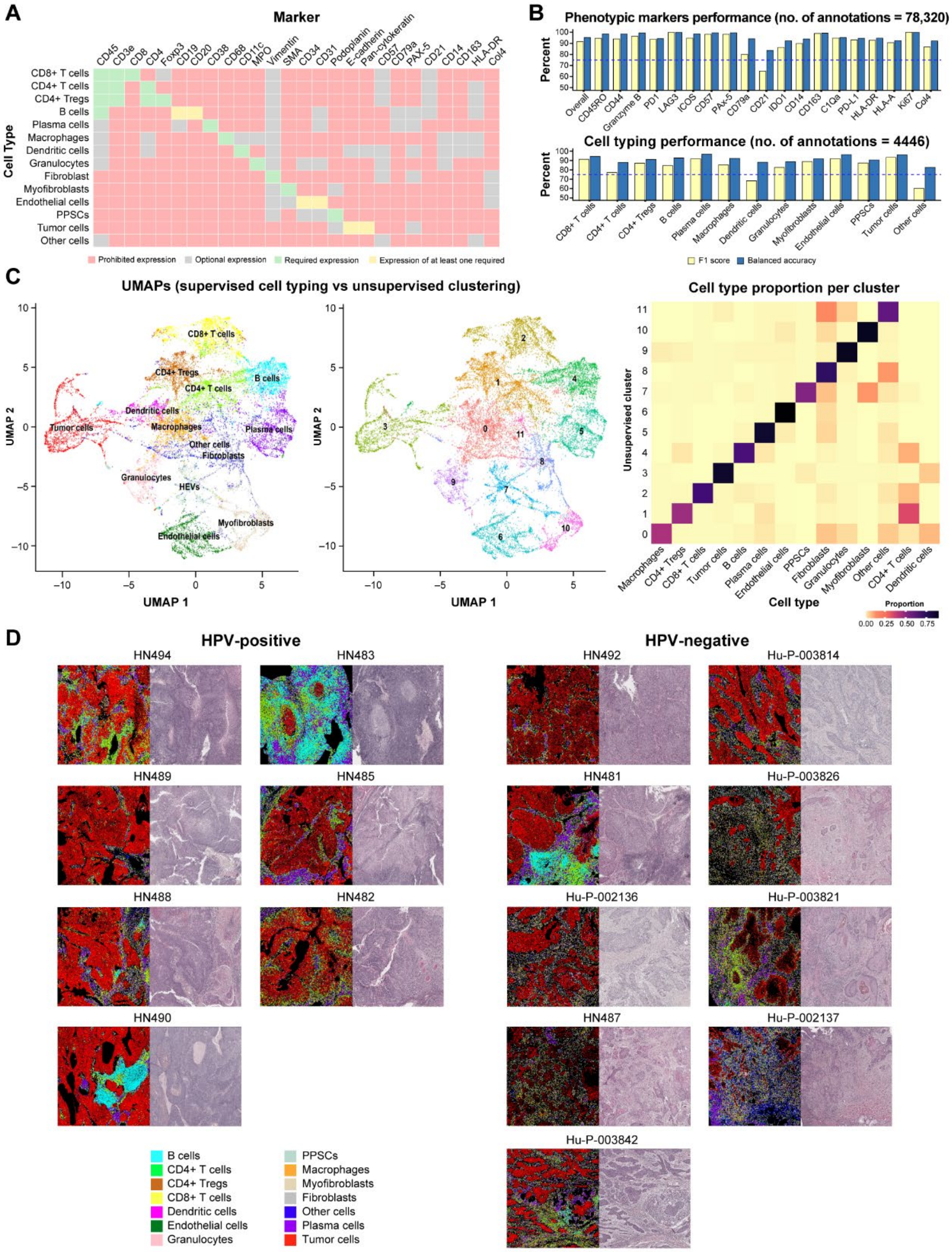

Suppl. Figure S1. Cell types exploration and performance evaluation of mIF analysis pipeline. (A) Cell typing protein expression matrix, defining which protein must be positive (green), either must be positive (yellow), must be negative (red), or can be either positive or negative (grey) for each cell type. (B) Phenotypic proteins and cell typing performance evaluation against manual annotations. (C) UMAP visualization (left) and heatmap of cell type fractions within unsupervised clusters (right), demonstrating high concordance between supervised cell typing and unsupervised clustering. (D) Representative images of mIF and H&E staining in HPV-positive and HPV-negative tumors, illustrating the spatial distribution of identified cell types across tumor regions.

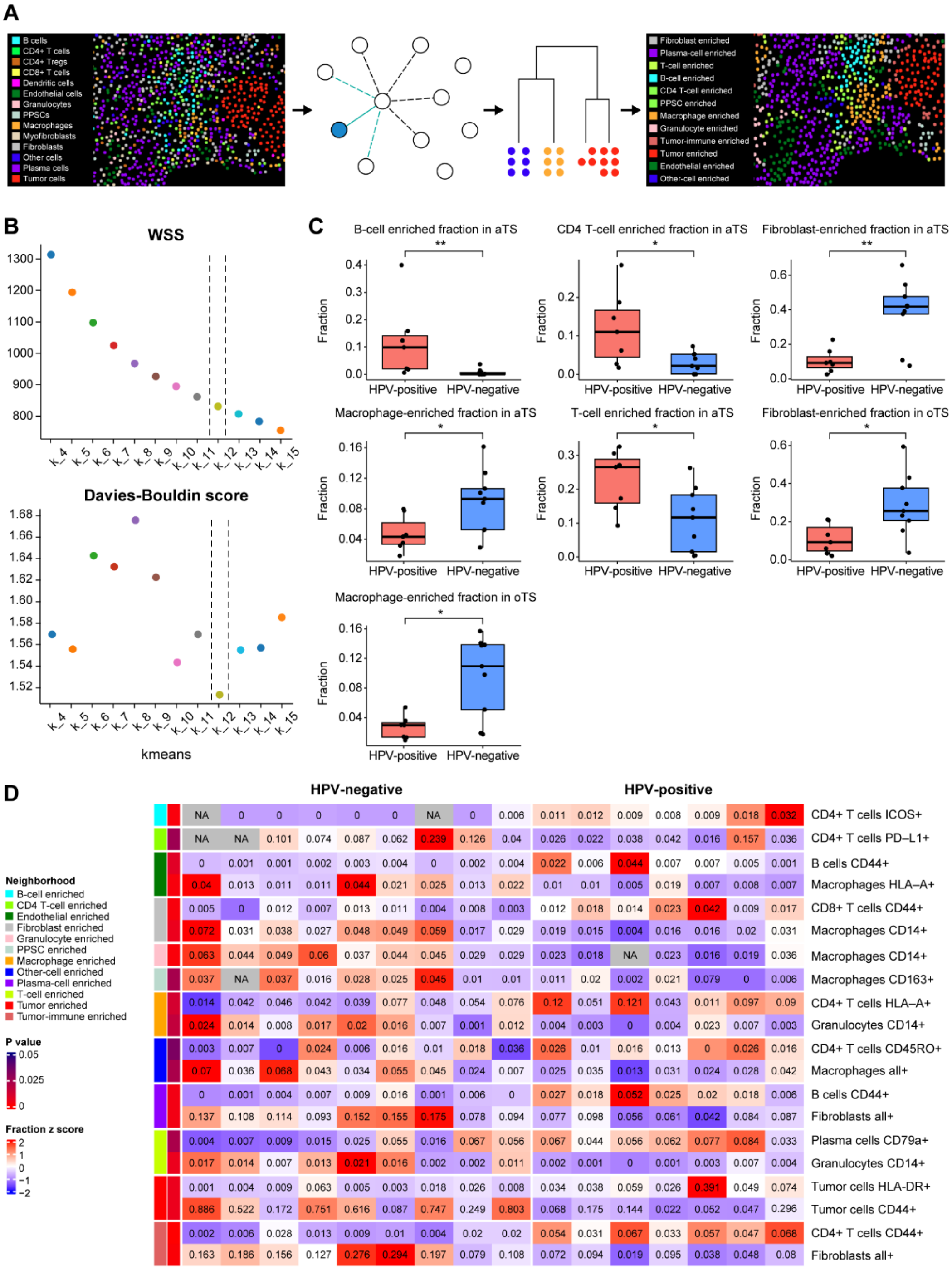

Suppl. Figure S2. Cellular neighborhoods identification and distribution between HPV-positive and HPV-negative tumors. (A) Schematic illustrating the identification of spatial cellular neighborhoods. (B) Plots showing within-cluster sum of squares (WSS) and Davies–Bouldin Index scores for different cluster numbers, used to determine the optimal number of spatial neighborhoods. (C) Box plots comparing the fractions of spatial neighborhoods in the tumor stroma of HPV-positive (n= 7) and HPV-negative (n= 9) tumors. (D) Heatmap of cell states distribution across neighborhoods, showing the top differentially expressed cell states (p<0.05) upregulated in HPV-positive (n= 7) and HPV-negative (n= 9) tumors. Heatmap cell colors represent fractions Z-scores and numbers indicate cell state fraction. P-values were calculated using the Mann-Whitney U test. * = p<0.05, ** = p<0.01.

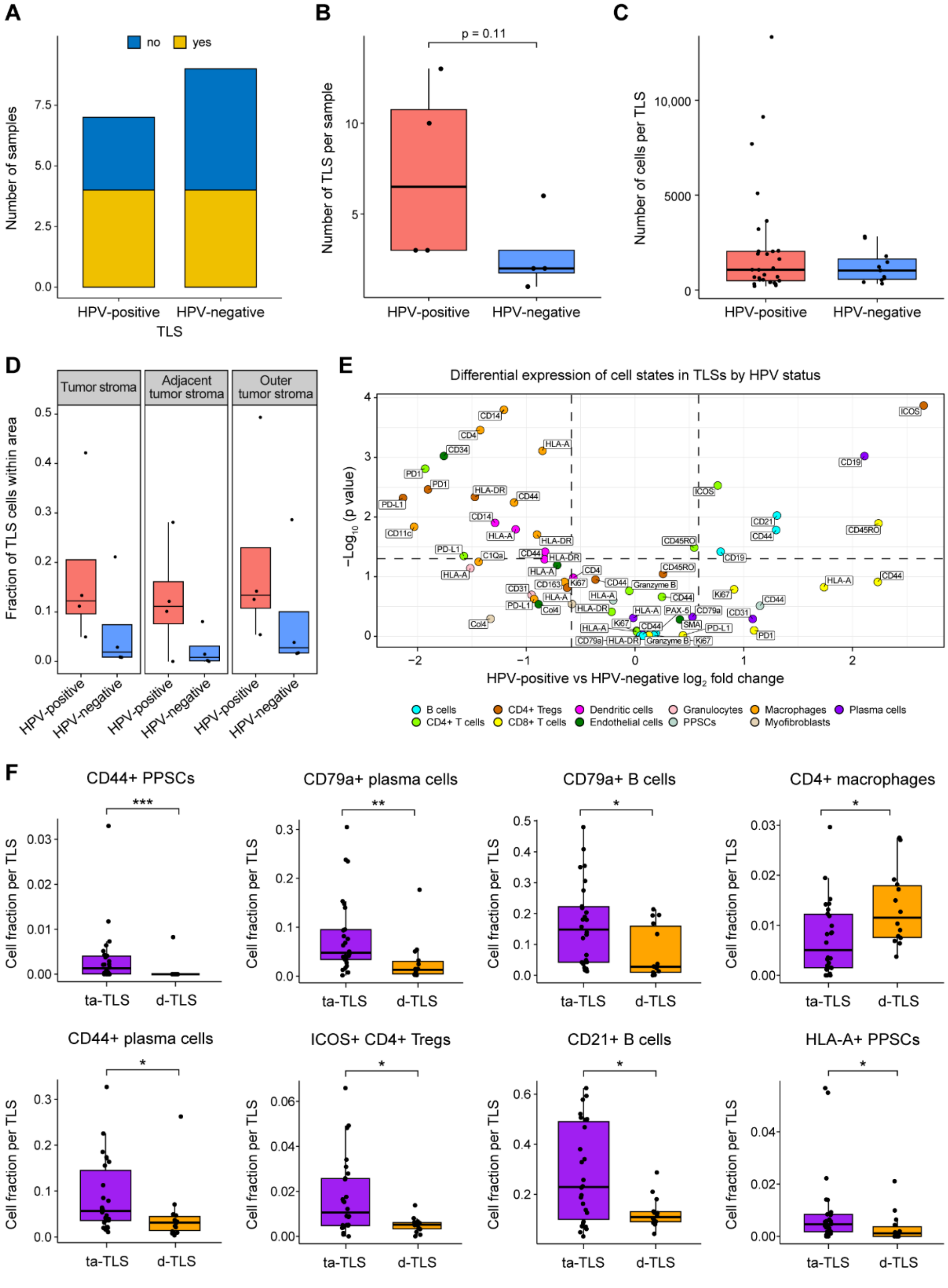

Suppl. Figure S3. Analysis of TLS by HPV status in HNSCC tumors. (A) Bar plot showing number of HPV-positive (n= 7) and HPV-negative (n= 9) samples with detected TLSs. (B) Box plot of the number of TLSs per sample. (C) Box plot of the number of cells per TLS, as an estimate of TLS size. (D) Box plots demonstrating the fraction of TLS cells from all cells within the tumor stroma, adjacent tumor stroma (aTS), and outer tumor stroma (oTS) by HPV status (HPV-positive; n= 7 and HPV-negative; n= 9). (E) Differential expression of cell states in TLSs by HPV status. Dot color represents cell type, and label represents phenotypic protein positivity. (F) Box plots of differentially expressed cell states between tumor-adjacent (ta-TLS; n= 26), and distant TLS (d-TLS; n= 14). P-values were calculated using the Mann-Whitney U test. * = p<0.05, ** = p<0.01, *** = p < 0.001.

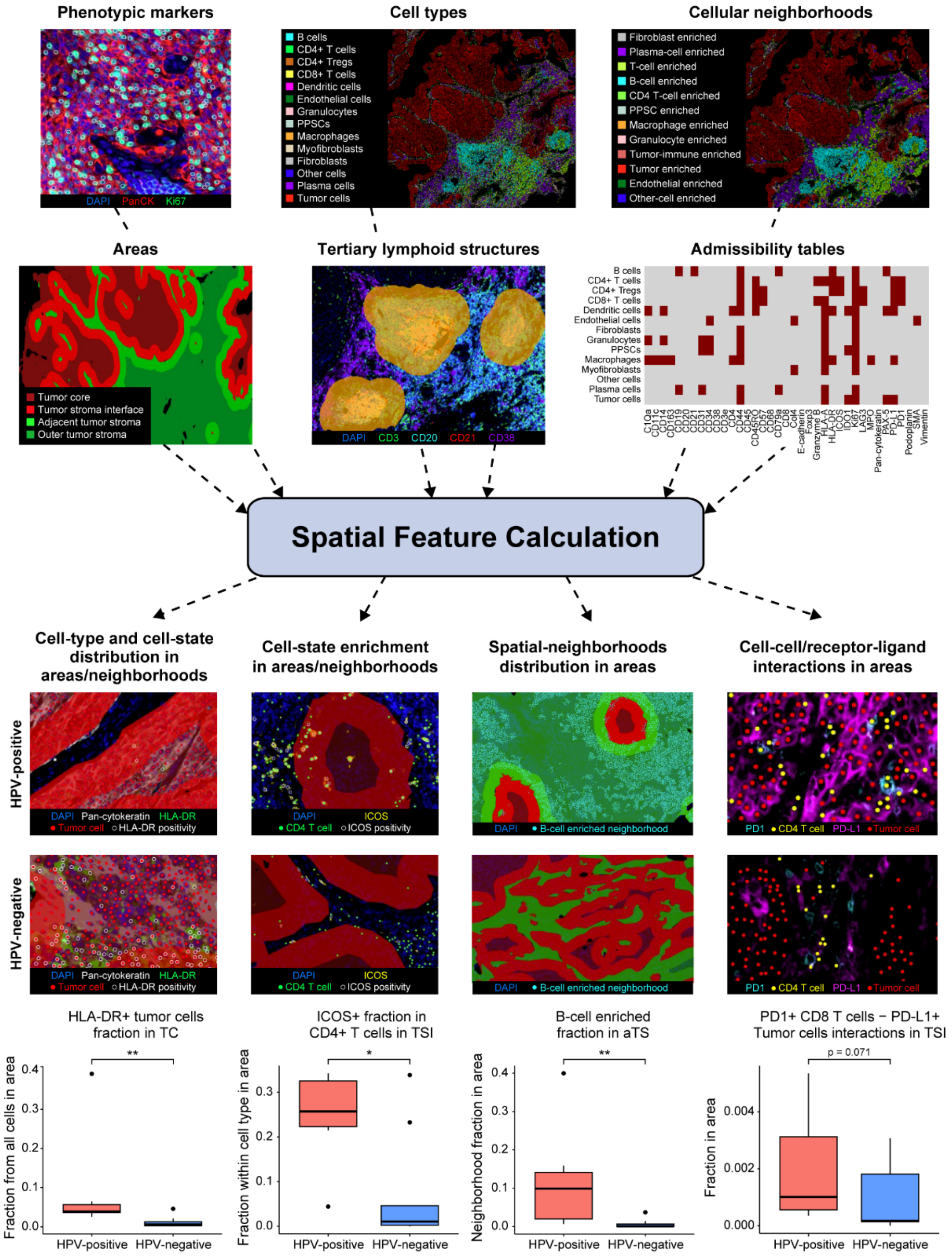

Suppl. Figure S4. Methodology for spatial feature calculation from spatially resolved protein data. Inputs consist of cell types, cell states, spatial neighborhoods, and tumor areas, including tertiary lymphoid structures (TLS). Admissibility tables guide the calculation by focusing on biologically relevant combinations of cell markers, areas, and neighborhoods. Spatial feature families include cell-type and cell-state distribution within neighborhoods/areas, cell-state enrichment, spatial-neighborhood distribution, and cell-cell/receptor-ligand interactions. Representative features from each family are shown through images and box plots comparing HPV-positive and HPV-negative tumors. P-values calculated using the Mann-Whitney U test. * = p<0.05, ** = p<0.01. TC (tumor core); TSI (tumor-stroma interface); aTS (adjacent tumor stroma); oTS (outer tumor stroma).

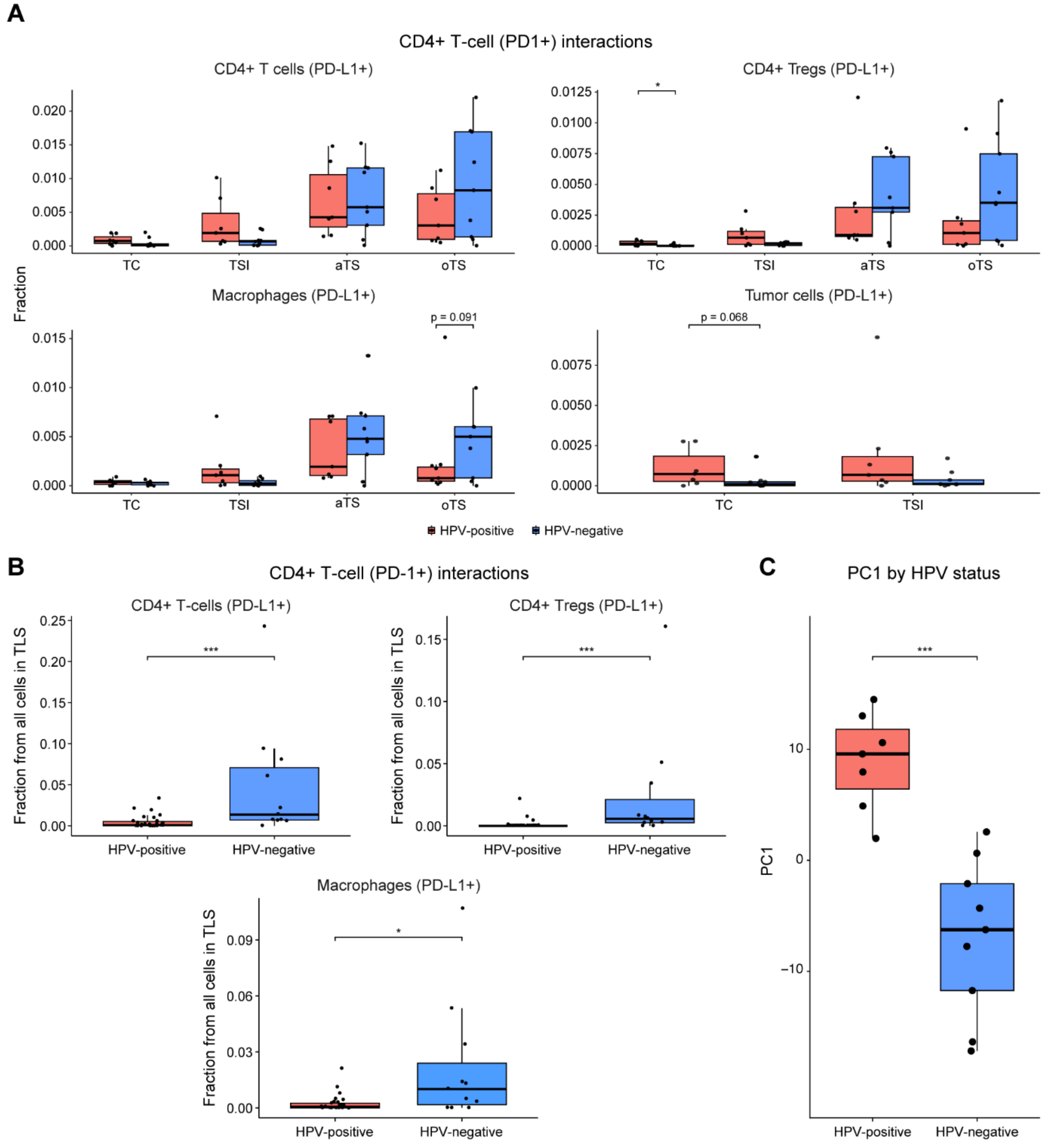

Suppl. Figure S5. Cell interactions and primary component distribution by HPV status. (A) Box plots demonstrating differential interaction frequency between PD-1^{+} CD4^{+} T-cells and PD-L1^{+} cells, in TC (tumor core), TSI (tumor-stroma interface), aTS (adjacent tumor stroma), oTS (outer tumor stroma) areas of HPV-positive (n= 7) and HPV-negative (n= 9) tumors. (B) Box plots demonstrating differential interaction frequency between PD-1^{+} CD4^{+} T-cells and PD-L1^{+} cells in TLS microdomain of HPV-positive (n= 7) and HPV-negative (n=9). (C) Box plot of Principal Component 1 (PC1) scores by HPV status. P-values were calculated using the Mann-Whitney U test. * = $p<0.05$, *** = $p < 0.001$.

Supplementary Tables:

ID	HPV status	Anatomical site	Sex	Age (years)	TNM stage			Grade	Therapy
					T	N	M		
1	Negative	Pharynx	M	47	2	3	0	2	Surgery
2	Negative	Larynx	M	49	3	0	0	2	Surgery
3	Negative	Larynx	M	68	4A	0	0	1	Other
4	Negative	Larynx	M	65	X	0	0	1	Surgery
5	Negative	Larynx	M	56	4A	0	0	1-2	Surgery
6	Negative	Larynx	M	62	X	X	0	2	Surgery
7	Negative	Oropharynx	M	67	4B	2C	0	2	Palliative radiotherapy
8	Negative	Pharynx	M	72	1	2B	0	2	Radiotherapy
9	Negative	Pharynx	F	69	2	0	0	1	Radiotherapy
10	Positive	Tongue	F	54	1	1	0	1-2	Chemoradiotherapy
11	Positive	Tonsil	M	42	2	1	0	1-2	Surgery and chemoradiotherapy
12	Positive	Tongue	M	74	1	1	0	2	Radiotherapy
13	Positive	Tonsil	F	48	1	1	0	1-2	Surgery and radiotherapy
14	Positive	Oropharynx	M	73	4	1	0	1-2	Radiotherapy
15	Positive	Tongue	F	78	2	0	0	1	Radiotherapy
16	Positive	Tonsil	M	56	3	1	0	2	Chemoradiotherapy

Suppl. Table S1 - Patient Characteristics

List of Additional Supplementary Tables:

Suppl. Table S2 - Cell rules and admissibility tables

Suppl. Table S3 - Cell types and cell states distribution in areas

Suppl. Table S4 - Neighborhoods analysis

Suppl. Table S5 - TLS analysis

Suppl. Table S6 - Cell interactions analysis

Supplementary References:

1 Niyati Jhaveri BBC, Nadezhda Nikulina, Ning Ma, Dmytro Klymyshyn, James DeRosa, Ritu Mihani, Aditya Pratapa, Yasmin Kassim, Sidharth Bommakanti, Olive Shang, Shannon Berry, Nicholas Ihley, Michael McLane, Yan He, Yi Zheng, James Monkman, Caroline Cooper, Ken O'Byrne, Bhaskar Anand, Michael Prater, Subham Basu, Brett G.M. Hughes, Arutha Kulasinghe and Oliver Braubach. Mapping the Spatial Proteome of Head and Neck Tumors: Key Immune Mediators and Metabolic Determinants in the Tumor Microenvironment. GEN Biotechnology 2023; 2 (5).

2 Ettai Markovits TD, Roman Gluskin, Ido Weiss, Amit Gutwillig, Tomer Dicker, Sun Dagan, Ron Elran, Becky Arbiv, Yuval Shachaf, Amit Bart, Assaf Debby, Nethanel Asher, Guy Ben-Betzalel, Ronnie Shapira-Frommer, Iris Barshack, Ori Zelichov. A novel deep learning pipeline for cell typing and phenotypic marker quantification in multiplex imaging. BioRxiv 2022.

3 Ronneberger O, Fischer P, Brox T. U-Net: Convolutional Networks for Biomedical Image Segmentation. 2015. [Epub ahead of print] doi:10.48550/arXiv.1505.04597

4 Schurch CM, Bhate SS, Barlow GL et al. Coordinated Cellular Neighborhoods Orchestrate Antitumoral Immunity at the Colorectal Cancer Invasive Front. Cell 2020; 182 (5): 1341-1359 e1319.

5 Davies DL, Bouldin DW. A cluster separation measure. IEEE Trans Pattern Anal Mach Intell 1979; 1 (2): 224-227.
